# Identifying communication spillovers in lab-in-the-field experiments^☆^

**DOI:** 10.1016/j.jdeveco.2022.102845

**Published:** 2022-06

**Authors:** Alexander Coutts

**Affiliations:** Schulich School of Business, York University, 4700 Keele Street, Toronto, Ontario, Canada M3J 1P3; NOVAFRICA, Portugal

**Keywords:** Lab-in-the-field, Public goods games, Field experiments, Development, Rwanda, Communication, Spillovers, SUTVA, Social learning

## Abstract

Use of lab-in-the-field experiments has steadily increased, given benefits of studying relevant populations and their preferences. In the field, researchers must often relinquish the control of a standard laboratory, raising the specter of communication from past to future participants. Little is known about the consequences of such spillovers, and recent literature indicates variation in how authors deal with them. I provide estimates of communication spillovers using existing data from public goods games in Rwanda, leveraging variation in planning the sequence of visiting 147 villages. The resulting order created opportunities for some villages to communicate with past participants. Using ex-post matching of villages with and without these opportunities I find that communication led to substantial increases in cooperation, suggesting that unanticipated spillovers can bias inference. I conclude with advice for creating protocols to deal with communication spillovers.

## Highlights

• Lab-in-the-field experiments are becoming increasingly popular research tools.
• These settings can involve risks of communication spillovers across sessions.
• I estimate spillovers across Rwandan villages, finding they cause significant bias.
• Reviews of the literature suggest variation in how authors deal with them.
• I conclude with practical advice and recommendations for mitigation.

*“Collusion/Contagion: Several members of our team have experienced some serious collusion among players in large villages where they have run significant numbers of experiments. The degree of collusion was extreme in all cases in spite of the fact that offers were anonymous.”*

- Roots of Human Sociality, Experiments in 15 Small-Scale Societies: Phase II Game Protocol

## 1 Introduction

Laboratory experiments to measure preferences and behavior have become a standard component of the economist’s toolkit, and now a significant number of lab experiments are conducted in the field: outside of a university classroom or computer lab, and with non-student populations (Gneezy and Imas, 2017). The utilization of such lab-in-the-field experiments has been growing in applied social science research. In development economics, researchers are putting more weight on the importance of understanding relationships between preferences and development outcomes. Such work is increasingly finding outlets in leading journals of economics and political science.^1^

The internal validity of such studies hinges on unbiased identification of preferences, which requires “no interference between units” (Cox, 1958 p.19), which is central to the “stable unit-treatment value

assumption” (SUTVA) (Rubin, 1980, Imbens and Rubin, 2015). The quote at the beginning of this paper suggests one prominent failure of SUTVA in the context of lab-in-the-field experiments: past participants communicating with and altering the behavior of future participants.^2^ Taken from the phase II protocol of a large scale project to conduct behavioral experiments in 15 “small-scale societies”, the investigators reference contagion from communication spillovers in earlier experiments. This earlier work, see Henrich et al. (2001), is now some of the most highly cited work in economics using the lab-in-the-field methodology.^3^

I refer to such spillovers as inter-session communication spillovers, or simply communication spillovers for brevity. They occur whenever participants in an experiment share any aspects of their experience, advice, or interpretation of the experiment with anyone else who has not yet participated, but is connected with a future participant.^4^ In methodological accounts of lab-in-the-field studies, communication spillovers are not mentioned as confounds that researchers need to be aware of.^5^ While many researchers appear to use strategies to avoid such spillovers, such as implementing minimum distance rules, a review of the literature suggests that this is not universal.^6^ Moreover, given the lack of evidence, the extent to which spillovers may bias inference remains debateable: each author or editor’s opinion may inform how seriously they take the issue. Finally, even if mitigation strategies were universally deemed important, sometimes it may be infeasible to implement them. For example, one may wish to conduct a framed field experiment with members of a farmers cooperative, who all work in the same region.

I study a lab-in-the-field experiment in Rwanda that I helped coordinate, involving 147 rural villages visited over three months. Early in the implementation, we visited a village where all participants contributed the maximum amount. A woman later explained that she was friends with some of the women in a neighboring village, and one of her friends had participated in the same game only two days prior. Her friend advised her to contribute the maximum amount, and she had shared this information publicly before the team had arrived. This anecdote provided the impetus for this paper, which came to fruition with the insight that the human planning process generated plausibly exogenous variation in the order the villages were visited.

Specifically, the logistical planner for this study only had access to a pre-determined set of few village characteristics and was personally unfamiliar with the villages. Thus, his ordering could only be conditioned on a small and finite set of observables, and not on private information. I therefore use a propensity score matching strategy, which distinguishes villages according to whether or not they had opportunities to communicate with past participants, and then matches them on this finite set of observables. The resulting comparison is between villages which appear ex-ante identical to the planner, but for idiosyncratic reasons were either treated, i.e. they had neighbors which previously participated, or not. Having the full set of conditioning variables helps fulfill the key selection on observables assumption underlying matching techniques, that treatment is independent of outcomes, conditional on observables. This takes the guesswork out of which variables need be included in the propensity score.

Implementing this strategy, I find significant increases in cooperation among villages which are geographically proximate, defined by specified distance cut-offs, to other past participating villages. Cooperation increases by 11%–14% depending on the matching estimators used, while spillovers cease to be significant for distance cut-offs greater than or equal to 2.5 km. Beyond these aggregate impacts, I find evidence that these spillovers can also bias estimated treatment effects: an alternative version of the public goods game played in a second round significantly increased cooperation, but only among villages which were not susceptible to spillovers. Overall, I speculate that the explanation for these spillover effects involves communication shifting beliefs about levels of cooperation upward, and subsequently shifting contributions among those who cooperate conditionally. Indeed, there is suggestive evidence that the treatment effect is driven by those who are identified as conditional cooperators in the sample.^7^

These results present novel estimates in a lab-in-the-field context of unstructured communication on behavior and suggest that researchers have reason to be concerned. Existing evidence is scarce — most related are the studies of Cardenas and Carpenter (2005) and Bernal et al. (2016), which focus on within-group learning in common pool resource lab-in-the-field games. Despite the different focus on the impact of returning participants, their suggestive findings that even novice participants in groups with experienced players cooperate more resonates with the results of this paper.

The results and discussion of this paper lead to a number of practical suggestions for researchers involved with lab-in-the-field studies, which may be particularly susceptible to spillovers when they involve high salience and stakes. It is especially important to develop and later publish “no-contact” protocols, which mandate minimum distance between sessions and/or formal rules implementing sessions in parallel. Section 4 details these best practices, as well as guidance for how to proceed when they may be infeasible.

## 2 Context and empirical strategy

### 2.1 Context

The lab-in-the-field games were conducted as part of a broader impact evaluation of community health programs. In parallel with baseline surveys for that evaluation, public goods experiments were planned in 150 villages in the Rusizi district in Rwanda over a three month period in 2013.^8^ The public goods experiments followed a typical format. Twelve participants were selected at random from the baseline survey list, each endowed with 400 RWF (0.60 USD in 2013). Communication was not permitted during the game. In private, participants had to decide how much of their endowment to keep for themselves, and how much to give to a group fund, which would be multiplied by 3 and divided equally among all participants. Each village played two rounds: the first round was identical for all villages and is the focus of most of this paper’s analysis, while the second, introduced only after the first, potentially involved alternative versions (discussion is deferred until Section 3.2). Further details of all procedures and the participants are in Online Appendix A.

Of the 150 villages visited, three are dropped from the analysis as we were unable to find the full 12 participants, leading to a final sample of 147. The average level contributed to the group fund is 255 RWF, which is about 64% of the socially optimal level of contributing the maximum 400 RWF. This is on the higher end of contributions in public goods games, particularly given the relatively low marginal per capita return of 0.25 (Ledyard, 1995). As is typical in public goods experiments, the self-interested theoretical prediction of contributing zero is rejected.

### 2.2 Identifying communication spillovers

To identify the effects of communication I utilize a nearest-neighbor matching strategy that examines the cooperation of paired villages which are similar, apart from one dimension: whether they had opportunities to communicate with previous participants. I define a village as being vulnerable to communication spillovers when it is located near of any other neighboring village that *previously* participated.

One first issue that arises is to select the appropriate distance threshold defining ‘near’. To remain partially agnostic, I present matching results for varying distances from 1 km to 3 km (in 0.25 km increments). Distances at these two endpoints are unlikely to be appropriate due to mis-categorizing villages with past participating neighbors as not having any, and vice-versa.^9^ I focus most of the analysis on the interior distance of 1.75 km. This moderate distance has the advantage of creating sample-size balance between villages with and without neighbors - a key advantage for the matching analysis.^10^ After selecting the distance threshold, one possible threat to the identification of spillovers is if villages with past participating neighbors have more neighbors in general, and villages with more neighbors happen to be more (or less) cooperative. This concern is addressed by the primary matching strategies, as well as a supplementary exact matching strategy which only pairs villages with the same number of neighbors.

A second, and more complex issue for identification, is the determination of when villages participate, i.e. the order of village visits. For example, if villages had been visited in order from richest to poorest, those visited later would be both poorer and more likely to have past participating neighbors. Ideally, order would have been randomized to ensure that there are no aggregate differences between villages which had and did not have opportunities to communicate with past participants. However, in the current study this was not the case. Instead, a study planner observed a set of pre-study observable characteristics of the 150 villages, and had to determine an ordering. Rather than assume (erroneously) that the planner chose randomly, I assume instead that after conditioning on all of the pre-study variables observed by the planner, the unexplained variation in order generates precisely the randomness needed for identification.

In the current context, this assumption appears reasonable. The planner was not a local of the study area and did not have private information about the villages, beyond the pre-study observables. His knowledge of Rusizi district was based on familiarity with the 18 political sectors of the district, their locations, and whether they are accessible by paved roads.^11^ How did the planner choose the order? As would be expected, the primary concern of the planner was logistical convenience: choosing to visit villages located nearby one another at similar dates. Two additional concerns featured in his decisions. The first was to ensure difficult villages were spread evenly throughout the study: those that were located far from the study’s base location, and/or those that had large numbers of households, which required longer working hours. The second was that on some days, the planner decided to alter the ordering due to heavy rainfall, as some regions were more difficult to access than others, due to a lack of paved roads.

These features of the planning process help to introduce variation into the ordering. The first re-balances these characteristics among villages visited earlier and later, which may correlate with opportunities for potential communication. The second introduces random shocks which forced the planner to alter the order. Overall, while most of the planner’s strategy was based on logistical convenience, there is variation in visit order which cannot be explained in a deterministic way by all of the pre-study observable variables. In addition to these shocks, the fact that the planner lacked detailed information on the villages, but nonetheless had to make order decisions among villages which appeared similar from his perspective, means that necessarily some decisions would have to be arbitrary. The key strategy exploits this variation in the planner’s decision making to find villages which were identical based on available observables, but by chance, differed in whether or not they had neighbors who previously participated in the public goods games: i.e. had potential opportunities to communicate with past participants.

### 2.3 Matching strategy

As noted, this setting is amenable to using propensity score matching (Rosenbaum and Rubin, 1983) to estimate the causal effects of communication spillovers on behavior. This is because a key assumption of matching techniques, selection on observables (i.e. that after controlling for observables, treatment assignment is independent of the outcome of interest) is likely to be satisfied automatically. The reason is that order of visit could only be conditioned on a small set of observable variables known to the planner, *before* the games were conducted.^12^

**Fig. 1:**
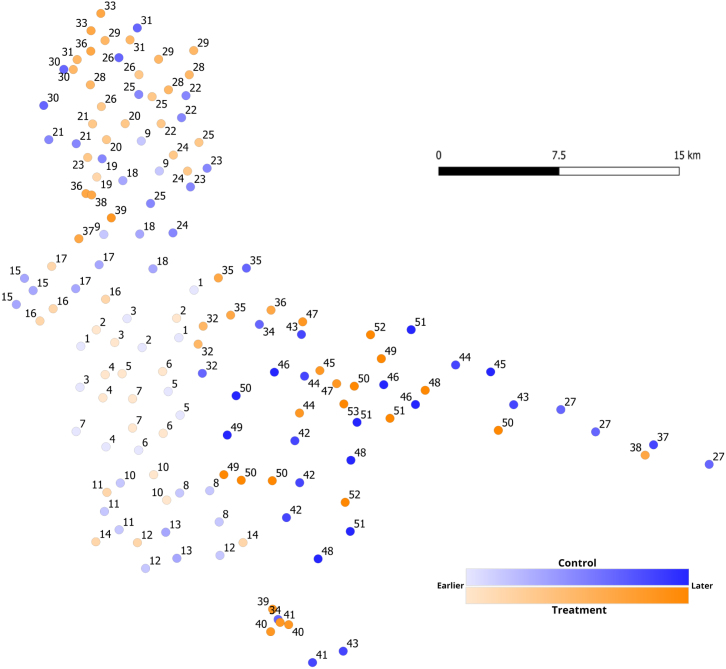
Treatment Status and Visit Order. Each circle represents one village. Each village is numbered according to its order of visit, and shaded accordingly. Thus villages with 1 are visited on the first day, while those with 53 are visited on the last day.

Villages are categorized as vulnerable to communication spillovers and hence *treated* if they had at least one neighboring village which previously participated in the public goods games. Otherwise, they are referred to as *control* villages. According to the main treatment definition of a neighbor as being located within 1.75 km, Fig. 1 shows the geographic dispersion and visit order of the resulting 73 treatment and 74 control villages.^13^

I now turn to outlining the key variables available to the planner. I will first look at the relationship with these variables and order of visit. Second, given that order determines treatment status, I will look at the relationship with these variables and treatment status. The key matching variables the planner had access to derive from the following: (1) distance to base (from village to study base location), (2) distance to paved road, (3) village size (number of households in the village), (4) number of total villages in the study located within 1.75 km (village density), and (5) sector.^14^ While these variables contain information which could be used in non-linear ways, it is important to note that a simple regression with the first four variables above entering linearly and sector fixed effects accounts for 93% of the variation in visit order, with fixed effects absorbing the bulk of this variation regarding village locations (Online Appendix B). This suggests that the planner proceeded more or less sector by sector, and that a simple linear weighting of these variables is sufficient to capture most of the planner’s decision making.^15^

Details of the relationship between the key matching variables and treatment status are relegated to Online Appendix C.1. The only significant correlation with treatment is variable (4) above, village density. This is not surprising, given that having neighbors is a pre-requisite for having neighbors that previously participated. Online Appendix C.1 also shows that there are no other pre-matching imbalances among other observables in treatment vs control villages, except one quite conspicuous one: average contributions in the public goods game. In particular contributions are 30 RWF *greater* in treatment villages, i.e. villages which had neighbors that were past participants.

Since treatment and control villages are similar on all observable characteristics related to demographics and preferences, one might in fact interpret the difference in contributions as the unbiased treatment effect, that is, the true impact of opportunities for communication on cooperation. However, propensity score matching will ensure that village differences in observables are controlled for in a more systematic way. Following the algorithm of Imbens (2015), the matching variables used to calculate the propensity score are the four variables corresponding to distance from base, distance from paved road, number of households in village, and village density within 1.75 km.^16^

## 3 Estimates of communication spillovers

### 3.1 Average treatment effect

To estimate communication spillovers, I focus on two main specifications for computing average treatment effects (ATEs) using the matching strategy outlined in the previous section and using two neighbors, with replacement. The first specification includes four of five key variables that the planner had available in the calculation of the propensity score, but does not include political sectors. To appropriately match on sector, the second specification uses the same four variables, but in addition, forces exact matching on political sector. Given that the planner’s knowledge was at the sector level, exact matching will control for any private information the planner could have about these sectors.

**Fig. 2:**
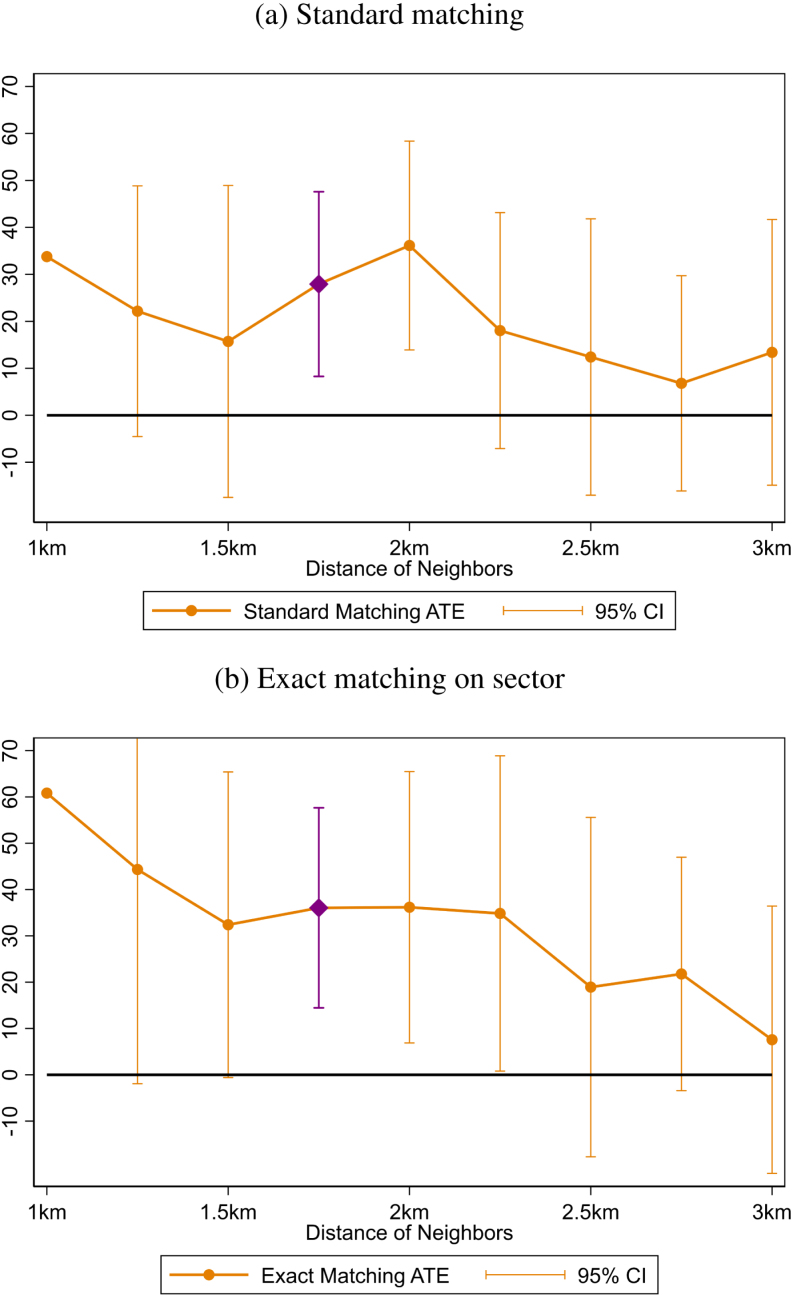
Matching estimates for different treatment cut-off distances. Each point corresponds to the average treatment effect (ATE) estimate for specified distance cut-off for independent nearest neighbor propensity score matching estimations (using 2 neighbors with replacement). Values of propensity score outside common support range are dropped. Exact matching excludes sectors with only 0 or 1 village in either treatment or control groups. Abadie–Imbens Robust Standard Errors are used to calculate error bars. Error bars suppressed for 1 km due to noise. Observations vary. Online Appendix C.3 provides the corresponding numerical estimates and their standard errors.

Fig. 2, Fig. 2 present these two key specifications for the main treatment (using a cut-off of 1.75 km) as well as alternative treatment definitions using distance cut-offs ranging from 1 to 3 km. Focusing on the main treatment which is showcased with a purple diamond marker, the ATE ranges from 27.9 (SE: 9.9; N: 118) to 36.1 (SE: 10.9; N: 102) RWF, each significant at the 1% level.^17^ These results are similar in magnitude, despite the specification differences, and correspond to a 11%–14% increase in contributions over the entire sample as a result of communication spillovers. Exact matching comes at the cost of fewer observations, but only a slight loss in match quality. Examining the full range of distance cut-offs, there is a pattern of shorter distances being associated with larger treatment effects, though there also arise issues of statistical power, due to few treatment villages when distances are shorter, and few control villages when distances are longer. Bearing in mind this caveat, for distance cut-offs of 2.5 km or more, estimated spillovers are no longer significant.

One potential concern highlighted earlier is the extent to which these estimates of increased cooperation are affected by villages with more neighbors more generally, as these villages are mechanically more likely to be in the treatment group. While matching is effective in balancing village density across treatment and control, one can go a step further and force exact matching on village density, an exercise that is done in Online Appendix D. The result for the main treatment is similar, 31.8 RWF, significant at the 1% level. Beyond this matching solution, OLS specifications also enable similarly spirited robustness checks, as well as alternative estimates of the main ATEs. It is reassuring that an analogous OLS specification of the main treatment estimates a similar effect of 28 RWF (Online Appendix E).^18^

Finally, the feature that the order of village visits determines treatment status in the above estimations can also be leveraged to generate a variety of counterfactual orderings, similar to the logic of (but not the same as) Fisher’s exact test. Consider one such example, of generating 10,000 counterfactual orders of village visits, chosen completely at random. For each order, one could define an analogous placebo treatment, and then estimate for each the corresponding ATE (or OLS) estimates of this paper. Of interest would be how often these estimates exceed those found here. In Online Appendix F I conduct stringent versions these types of tests, including one which requires that sectors are visited in the same order selected by the actual planner. That exercise holds constant the sector ordering the planner chose, but provides a measure of how “lucky” he would have needed to be, to by chance, order villages in such a way which generated the large treatment effects observed. The result of this exercise is that the main estimates in the paper exceed 99.9% and 99.2% of these derived estimates for standard and exact matching estimates respectively (for the main treatment 1.75 km). Taken together, these results suggest substantial evidence that indeed communication spillovers between villages significantly increased cooperation.

### 3.2 Spillovers across ‘treatment’ versions

The previous subsection provided evidence that communication spillovers led to significant increases in cooperation in these public goods games. However, many lab-in-the-field games are conducted with the aim of comparing two (or more) ‘treatment’ versions of a particular experiment.^19^ One might expect that, if communication spillovers have the same impact on two different experimental versions, then one can still derive the unbiased ‘treatment’ effect.

Unfortunately, the result of this paper suggest that is not guaranteed to be the case. In the current context, I utilize the fact that in the *second* round of the public goods games, villages either conducted the same baseline version again (52% of the sample) or conducted one of three alternate versions involving opportunities to reward, punish, or a game which involved aggregate risk. I pool these three alternative versions together.

**Fig. 3:**
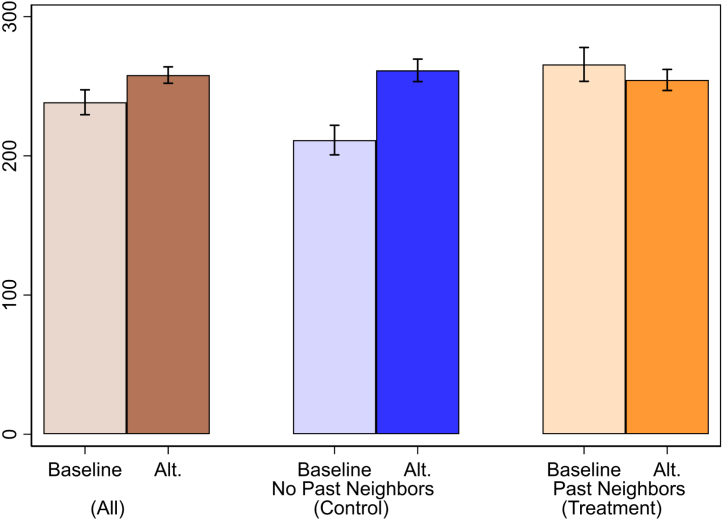
Contributions in Second Round by Treatment Status and Game Version. Average contributions in the *second* round, using fitted values from OLS regression interacting treatment status and game version. Baseline refers to the baseline game repeated. Alt. refers to an alternate version involving either: rewards, penalties, or aggregate risk. 95% confidence intervals shown, N=147 villages. See Online Appendix G for more details on game versions and the full set of OLS controls used.

Fig. 3 presents a summary of average contributions in the second round depending on whether the version was the baseline repeated or was one of the alternate versions, by treatment status (presence of past participating neighbors within 1.75 km). Villages which had no past participating neighbors (control), show significant increases in contributions between baseline and alternate versions. Those with past participating neighbors (treatment), show elevated contributions in both baseline and alternate versions, though critically, there is no significant difference across these versions.

While exploratory, these patterns suggest that communication spillovers can lead to differential impacts across ‘treatment’ versions, and hence bias these ‘treatment’ effects. Interpreting the control group as the counterfactual that would have resulted without communication spillovers, the alternative versions have a clear impact of increasing contributions. However, there is no analogous impact for the treatment group, and the estimated effect for the full sample is muted, and in fact not significant in a corresponding regression (p-value 0.215).^20^ Thus, spillovers may have crowded out the ‘treatment’ version effect, which otherwise made control villages more cooperative.

### 3.3 Mechanisms

It is worthwhile to investigate the potential mechanisms behind the observed increase in cooperation. This may shed light on the circumstances for when (or with what populations) we might worry about spillovers. Since communication was not observed directly, I rely on indirect inference to distinguish potential mechanisms. As such, this section should be viewed as exploratory in nature.

It is useful to start from the existing evidence from lab experiments on how communication alters contributions in public goods games. I focus on two phenomena: advice and pre-play communication. Regarding the former, Chaudhuri et al. (2006) demonstrated in public goods experiments that when past participants gave public advice to future participants, this advice led to significantly higher contributions.^21^ Regarding pre-play communication, Isaac and Walker (1988) documented a significant role of such communication in increasing contribution rates in public goods games.

In both cases, conditionally cooperative behavior (contributing more when expecting others will do the same) plays a central role. Chaudhuri et al. (2006) showed explicitly that conditional cooperators (CCs) were the driving force behind the increased contributions in their advice treatments. Regarding pre-play communication, absent social sanctioning such communication is cheap talk: any promises or announcements are non-binding. Yet lab evidence has found that individuals are often nonetheless honest in such settings, and that cheap talk can therefore increase cooperation when benefits are sufficiently high (Arechar et al., 2017). Unlike free-riders or those who cooperate unconditionally, CCs would be responsive to such communication.

I am unable to distinguish whether communication involved giving advice, other pre-play communication such as making promises, or even communication which took on a collusive tone.^22^ Nor is it reasonable to assume that every instance of communication followed the same structure. Instead, I examine whether there is evidence consistent with communication altering participants’ beliefs about cooperation in villages with past participating neighbors. To do so I first identify which subjects are most likely to be CCs, and next examine whether villages with more CCs are more likely to increase their contributions as a result of having neighbors who previously participated.^23^

As previously noted, individuals played a second round of the public goods game. As a proxy for being a CC, an individual is coded as such if they contribute in the second round the nearest allowable amount to the modal contribution in the first round. Online Appendix H examines OLS specifications which interact the main treatment dummy with this resulting variable for the proportion of CCs. In that specification, the effect of the main treatment disappears, while the continuous interaction is large and positively significant at the 5% level.

**Fig. 4:**
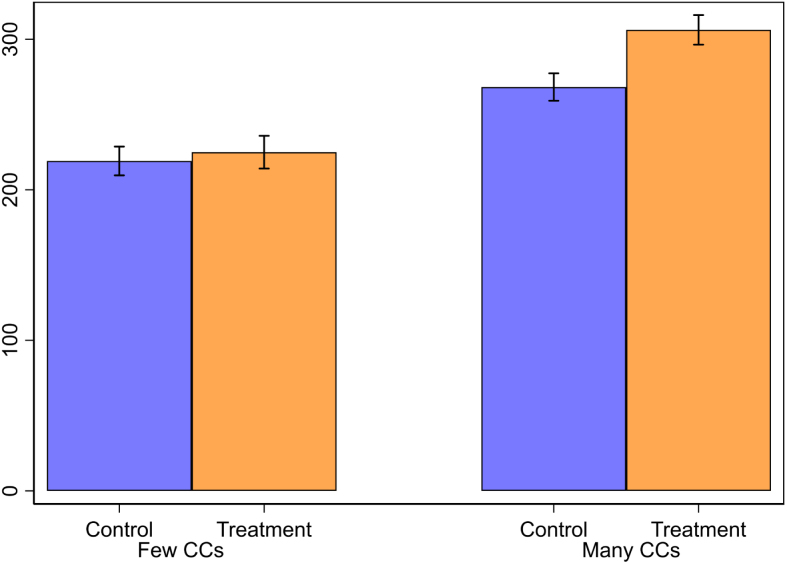
Heterogeneous Treatment Effects by Proportion of Conditional Cooperators. Average contributions, using fitted values from OLS regression interacting treatment status and proportion of CCs. Treatment and Control refer to whether the village had past participating neighbors or not, respectively. Proportion of CCs is categorized as few or many based on the sample median (27% of participants classified as being CCs). 95% confidence intervals shown, N=147 villages. See Online Appendix H for more details on classification of CCs and the full set of OLS controls used.

A summary of these aggregated heterogeneous treatment effects is shown in Fig. 4, where the proportion of CCs is instead split according to its median value of 27%. This figure showcases this significant interaction effect, showing that communication spillovers are only found in villages with many CCs, which are also more cooperative overall. This lends some support to the hypothesis that communication positively altered beliefs, with CCs driving the resulting positive increase in contribution rates among villages with past participating neighbors.^24^

One final related question is whether the specific outcome of previous neighboring games mattered for future participants. In theory the net impact of poor outcomes on beliefs is ambiguous. On one hand, poor outcomes may lead to pessimistic communication, lowering beliefs about overall cooperativeness. On the other hand, poor outcomes could motivate providing even more urgent advice to future participants to not make the same mistake, which could increase expectations of cooperation among recipients of advice. I can examine this question looking at the 73 treated villages with past participating neighbors, comparing contributions based on whether the nearest past participating neighboring village contributed above or below the median. The level of contributions is nearly identical: 270 and 269 RWF, respectively, with similar results found using OLS with all controls.

## 4 Discussion

Lab-in-the-field experiments have become increasingly important for research in economics and political science, as a means to study behavioral preferences and how they relate to broader economic outcomes. Yet field contexts may generate opportunities for communication between past and future participants, violating necessary conditions for unbiased identification of preferences. The results in this paper present evidence that communication took place and changed behavior in public goods games in the field, both in the aggregate, and in a way that altered the estimated impact of alternative ‘treatment’ versions.

How has the existing literature dealt with these concerns? Although not an exhaustive search, Online Appendix J provides details of 17 recent articles found in leading journals which involve lab-in-the-field studies, including 7 from the *Journal of Development Economics*. First, about two-thirds of these articles make no mention of spillovers, nor appear to address them in their experimental protocols. More concerning, a reader could be worried about potential spillovers for approximately half of them. To investigate more, I contacted all authors, a majority of which provided further information not available in the paper or supplementary material. Yet, after accounting for this, it remains that a reader could still harbor some concerns about inter-session communication spillovers for over one in four of these studies.

How should lab-in-the-field experiments be conducted to minimize the effects of unintended communication on outcomes, and what can be learned from potentially vulnerable studies? The first solution involves trying to identify whether communication is likely to be an issue in a given study. Two contexts appear particularly problematic. The first and most common, is that researchers conducted lab-in-the-field games repeatedly in the same location. The second is that researchers conducted them in rural communities which were located near other participating communities, as was the case for this paper. Underlying the likelihood of communication, are what I refer to as *plausible connections* among participants. Examples of such connections include participants who are members of the same farmers cooperative (Casaburi and Macchiavello, 2018), or simply neighboring households within or between villages. Clearly not all contexts will have plausibly connected participants: (Enos and Gidron, 2018) randomly approached subjects across cities in Israel, while (Jakiela and Ozier, 2015) ensured participating communities were 5 km apart.

Appendix A.1 describes plausible connections in further detail, as well as a range of solutions that can be adopted to prevent spillovers. The best solutions will ensure that such connections among participants are unlikely. Three strategies should become common practice. First, whenever possible, a strategy of minimum distance requirements (e.g. 5 km) should be pursued. Yet often, it is not possible to avoid conducting sessions in the same location/geographic area (or with a population with pre-existing connections). In this case, best practices would suggest a second strategy of running simultaneous parallel sessions to the extent possible. It may be infeasible to conduct all sessions at the same time: following a third strategy, one can restrict contact between sequential sessions by requiring participants to queue in a controlled waiting room, and only enter the session after the previous participants have left (D’Exelle and Verschoor, 2015).^25^ I refer to these three strategies as no-contact protocols, noting that they are not mutually exclusive.

Returning to the 17 studies reviewed, many involve smaller clusters of connected participants. Here, best practices would be to (ex-ante) identify and partition participants into precisely those sub-groups which are plausibly connected (e.g. by neighborhood, school district, proximity to other villages), followed by implementing the no-contact protocols outlined above. For example, Czura (2015) conducted lab-in-the-field games with participants at one location, a central MFI office — but participants coming from the same borrowing centers were typically transported in groups which then participated in the same sessions. At the same time, it is also clear that in some cases it is simply not possible to implement these no-contact protocols with a given population. In this case, there is not an obvious set of best practices to follow. The protocol of Kosfeld and Rustagi (2015) presents a potential option: for connected participants, ensure that sequential sessions are conducted as rapidly as possible, within the same day.^26^ However, these solutions may not completely exclude spillovers: as such, one should randomize and record session order within each sub-group to perform ex-post tests for spillovers.

**Above all, authors should create, commit to, and report no-contact protocols.** A transparent way of ensuring this is to include such protocols in pre-analysis plans. Only a minority of the reviewed studies included details about such protocols, and while most had some (often informal) strategies designed to minimize communication spillovers, rarely did it appear that they were applied systematically, and never does it appear that any ex-post tests of spillovers were conducted. These simple changes to how researchers approach lab-in-the-field experiments can make a large difference, both in eliminating the potential of such spillovers to bias results, but also for increasing transparency. This will discipline existing work, and help to establish stronger reporting norms, which will strengthen future work.

The results of this paper have shown the potential consequences: communication can substantially bias behavioral estimates. In the present case, communication had positive impacts on cooperation, with suggestive evidence that this was driven by the presence of conditionally cooperative individuals. Yet generalizability may depend on numerous factors such as the nature of the game, and local norms. As proof of this, the editor handling this paper has reported finding a negative and significant decline over time in contributions across public goods games held in the same location within groups of borrowers in a microcredit program. Thus, while the current paper illuminates some of the mechanisms at work, there remains much to understand about the black box of communication spillovers. For now, researchers should take greater care in designing and reporting protocols to avoid them, with the hope that future research will bring more insight towards developing evidence-based mitigation strategies.

## Data Availability

Data will be made available on request.

## Appendix A

### A.1 Plausible connections and spillovers

In identifying whether communication spillovers have the potential to impact a given study, it is useful to consider *plausible connections* among participants. These connections may be common to all participants, but more likely, they may exist for smaller subgroups of participants.

**Some examples of plausible connections:**

• Colleagues at work/school (Parents: children are classmates).
• Members of common association/cooperative/institution/community group.
• Urban/semi-urban neighbors: Living on same street (high density), living in same neighborhood (low density)
• Rural neighbors: Living within 5 km of one another.^27^
• Other context specific connections.

### A.2 No-contact protocols to minimize inter-session communication spillovers

Below I outline a short guide for how to proceed to minimize spillovers in a research design. There are often trade-offs involved. For example, ensuring 5 km between sessions in a rural area may substantially increase logistical costs. Beyond this, there may be trade-offs related to the population of interest. For example, trying to find different sub-groups which are not connected may require one to include less similar populations in the sample. These are difficult trade-offs, and I could not hope to provide useful advice for how to navigate them.

Assuming for simplicity that these trade-offs do not exist, the first step is to ask the following question:

**Are there likely to be some members of the population of interest of the study who are plausibly connected?**

If no, it is sufficient to record why this is the case, and ensure it is reported in any final work. As their is low likelihood of communication spillovers, no specific protocols need to be used.

If yes, the next step is to partition the population into greatest number of non-connected subgroups. Conduct parallel sessions with each subgroup. If not possible to conduct all sessions in parallel, ensure no contact between sessions using waiting rooms. If it is not possible to guarantee no contact, either because it is infeasible to completely partition participants into non-connected subgroups and/or run parallel sessions, then spillovers are possible. Best practices are to conduct sessions as rapidly as possible within the same day (Kosfeld and Rustagi, 2015), or following the preliminary evidence of Online Appendix I, to have a cooling off period of at least 7 days. If possible, randomize and record session order within each nonconnected sub-group to perform ex-post test for spillovers. It is also recommended to explicitly survey participants to examine whether they have had previous contact with others about the activity.^28^

Below are some examples of different scenarios and how one can best avoid potential communication spillovers from past to future participants.

*Example 1: Residents of rural villages in Rwanda:*

First best: Minimum distance of 5 km between all participating villages. Second best: Parallel/no contact sessions for all participating villages within 5 km of one-another. Third best: Risk of spillovers. Randomize order within each cluster of plausibly connected villages and conduct sessions either (i) as rapidly as possible, or (ii) with a cooling-off period of at least 7 days.

*Example 2: Example: Members of farmers cooperative in India:*

First best: Parallel/no contact sessions with all participants. Second best: Risk of spillovers. Randomize order and conduct sessions either (i) as rapidly as possible, or (ii) with a cooling-off period of at least 7 days.

*Example 3: Example: Residents of city in Israel:*

First best: Parallel/no contact sessions with all participants. Second best: Partition plausibly connected participants into the same session, such that there is no/low probability of contact across sessions. Third best: Risk of spillovers. Randomize order and conduct sessions either (i) as rapidly as possible, or (ii) with a cooling-off period of at least 7 days.

*Example 4: Example: Residents of one rural village in Portugal:*

First best: Parallel/no contact sessions with all participants. Second best: Risk of spillovers. Randomize order and conduct sessions either (i) as rapidly as possible, or (ii) with a cooling-off period of at least 7 days.

## Appendix B Supplementary data

MMC S1 .

## Footnotes

1 Recent examples published in top journals include Avdeenko and Gilligan (2015), Bauer et al. (2018), Casaburi and Macchiavello (2018), Chandrasekhar et al. (2018), D’Exelle and Verschoor (2015), Enos and Gidron (2018), Gneezy et al. (2016), Jakiela and Ozier (2015), Kosfeld and Rustagi (2015), and Somville and Vandewalle (2018). Use of lab-in-the-field experiments is also on the rise in other fields such as sociology, e.g. Baldassarri (2015)..

2 These concepts are typically framed in the context of ensuring unbiased comparisons between treatment and control groups. However it is straightforward to translate them to a unit group context, e.g. population choices in lab-in-the-field experiments. Using the classic SUTVA language, denote participation as “treatment”. Hence every participant is treated, and SUTVA requires that outcomes depend only on individual treatment, and *not* on the treatment of others. The assumption of SUTVA thus requires that the participation of others is unrelated to one’s choices. Communication from past participants can also be interpreted as a violating the requirement of excludability (Gerber and Green, 2012), which fails when some participants enter the game with different preparation that impacts choices: e.g. if some participants receive worse explanations of the game than others, or (as in this paper) if some participants have communicated with others prior to participation.

3 The protocol can be found here: http://www.ensminger.caltech.edu/documents/587/3_-_Game-Procedures-and-Protocols-DG-UG-TPP.pdf. In the protocol they discuss mitigation strategies, but conclude that the spillovers are impossible to avoid altogether. One may be concerned if prevalence of spillovers is correlated with group characteristics such as “market-integration”.

4 In the context of lab experiments: (Schotter, 2003) shows how advice changes behavior, Chaudhuri et al. (2006) demonstrates how beliefs about others can alter own behavior, and Zizzo (2010) and Dufwenberg et al. (2011) show how framing and experimenter demand effects can affect behavior.

5 Neither (Harrison and List, 2004) nor (Gneezy and Imas, 2017) discuss spillovers as a potential threat to the validity of lab-in-the-field studies. Viceisza (2012) does not discuss spillovers directly, but while discussing sampling recommends the scattering of households, subject to “reasonable limits”, which are not further defined.

6 Many lab-in-the-field experiments do not report timing and location of sessions, or whether any protocols were implemented to prevent spillovers, in order for readers to assess the degree to which they should be concerned. In Section 4 of this paper I briefly review a subset of the literature, including the publications mentioned in the first footnote, as well as recent papers from the *Journal of Development Economics*. I find that the majority of papers do not mention spillovers, nor protocols to avoid them. After contacting the authors for further details, reasonable concerns with spillovers were a feature in over one in four of the surveyed papers. Even if we assume adequate care is typically taken, we still lack the evidence to comment on both the magnitude of the problem we are taking care to avoid, and the effectiveness of potential mitigation strategies, such as ensuring minimum distances between sessions, or running sessions in parallel.

7 These results are consistent with (Chaudhuri et al., 2006), who find that providing future participants with public advice from past participants in a public goods lab experiment increases average contributions.

8 The broader research project name is: Impact Evaluation of Community-Based Health Programs in Rwanda (CBEHPP); ClinicalTrials.gov Identifier: NCT01836731; PI: James Habyarimana, Georgetown University. See Sinharoy et al., 2016, Sinharoy et al., 2017. As the evaluation found no impact of the health programs, the games were considered no longer essential for the final analysis, and hence were not utilized in these studies. For the purposes of this paper, the evaluation of the community health programs is not relevant, as these programs were implemented after the completion of all the public goods experiments.

9 Using the 1 km threshold, 90% of villages have no past participating neighbors. For 3 km there is the opposite issue: 80% of villages are categorized as having at least one past participating neighbor.

10 The first draft of this paper chose the distance of 2 km as an intuitive starting point. A subsequent draft, Coutts (2019), presents an earlier version of the main results of this paper and its appendices for both 1.75 km and 2 km. It is also worthwhile to note that implicit in the definition of a village being categorized as vulnerable to communication spillovers is that the threshold for this vulnerability is one neighbor (and not e.g. 2 or 3), and that the village is vulnerable if a neighbor has participated at any point in the past (and not e.g. only if that neighbor participated within the last 7 days). To the extent that the definition used may mis-categorize villages, this should work to bias the estimated spillovers towards zero. In supplemental analysis, I examine heterogeneous treatment effects by days elapsed since neighbors participated, to shed light on the this issue empirically.

11 Figure A1 in the Online Appendix presents a map of these sectors. The planner was interviewed in October 2019 by the author.

12 In most empirical studies, this assumption (also referred to as unconfoundedness) is difficult to satisfy, since often the treatment of interest, e.g. job training, is self-selected into by individuals, and the econometrician may not observe all relevant variables that determine this selection.

13 Out of the 73 treatment villages, 75% have exactly one neighbor that previously participated; 15% have two neighbors, while the remaining treatment villages have between three and five past-participating neighbors.

14 It is not possible to match on location, however distance to the base location and to a paved road conveys similar information, as does the addition of political sectors.

15 This is not to say that the resulting order was entirely predictable. Online Appendix B also presents information on the distribution of residuals from this simple regression.

16 Details about this algorithm are found in Online Appendix C.2, along with the distribution of the propensity score by treatment status. There is broad overlap across treatment and control villages, and the analysis will further restrict matching to occur in regions with common support.

17 Online Appendix C.3 provides further details for each of the 18 matching estimates shown in Fig. 2, Fig. 2. Online Appendix C.4 provides more details for the main treatment about post-matching covariate balance and alternative matching specifications that use different number of neighbors.

18 The validity of the unconfoundedness assumption that underlies the matching strategy is also required for unbiased OLS estimation, along with a further assumption of linearity of the conditional expectations of outcomes (Imbens and Wooldridge, 2009). The use of OLS also enables a number of useful specifications that can be used to define alternative dependent variables to capture opportunities for communication, and to run placebo style regressions and other robustness exercises. These specifications are examined in Online Appendix E.

19 The word treatment is placed between single quotations to distinguish its use here from that of other sections.

20 Online Appendix G presents the corresponding OLS specification with all controls, analysis of the three alternative versions separately, and further details on the versions themselves.

21 Note that there are differences between their controlled setting and this field experiment. First, their subjects are incentivized to provide advice, as they receive a portion of the future subjects’ earnings. Second, in the common knowledge treatment where they find the largest effects, advice is read out loud in front of all participants.

22 Since individual decisions were completely anonymous, explicit collusive arrangements can be ruled out. However, since the identity of those who played was not anonymous, individuals could make inferences based on the outcomes and priors about the cooperativeness of other participants. In this sense reputation and/or fear of reprisal could still operate indirectly. I thank an anonymous referee for highlighting this point.

23 It is important to re-emphasize the exploratory nature of this exercise for two reasons. First, classification of CCs must necessarily be done ex-post, given that the strategy method was not utilized (Selten, 1967). Second, the proportion of CCs in each village could itself be affected by communication, rendering it a “bad control” using the language of Angrist and Pischke (2008).

24 Online Appendix H provides more background on mechanisms more generally, and presents further details and robustness checks for the definition used for CCs. Other potential mechanisms ruled-out in that section include the possibility that communication changed cooperative norms, or that cooperation evolved over time (e.g. as a result of changing enumerator behavior). An earlier version of this paper showed no other heterogeneous effects besides those found for CCs (Coutts, 2019).

25 While this works for two sequential sessions, one could imagine other strategies to keep past participants occupied with another task or to have a second waiting room for a third group of participants. This part of the protocol of D’Exelle and Verschoor (2015) was detailed in private correspondence.

26 Exploratory results in Online Appendix I suggest that an alternative could be waiting at least 7 days between sessions with connected participants. Critically, there is no solid empirical evidence on the efficacy of either strategy: a methodological contribution would be to test various delays (e.g. same day, 3, 7, or 14 days).
