# Supplementary material for: Identifying communication spillovers in lab-in-the-field experiments
Source: J Dev Econ. 2022 Jun;157:102845. doi: 10.1016/j.jdeveco.2022.102845 (PMC9193284; doi:10.1016/j.jdeveco.2022.102845)
Supplement: MMC S1 — . [file mmc1.pdf]

Online Appendix for: Identifying communication spillovers in lab-in-the-field experiments

Alexander Coutts*

January 2022

A Protocol, Summary Statistics, and Scripts

A.1 Protocol

In total 150 villages were randomly selected to participate in the broader health evaluation: Impact Evaluation of Community-Based Health Programs in Rwanda (Sinharoy et al., 2016, 2017), and hence in the public goods games. These villages were chosen randomly from a total of 598 villages in the district, which consists of the 18 political sectors shown in Figure A1. The public goods games were conducted in parallel with baseline surveys. Regarding individual selection for participation in the games, 12 individuals were chosen randomly from the baseline survey list, which included one person from every household in a selected village with at least one child under the age of 5, and were given a ticket to participate in the games the following working day. The team typically visited 2 or 3 villages per day. The average visit time of 3 hours included time spent tracking down the selected participants. At the time of the games, the 12 individuals were checked-in by the survey team and completed a brief questionnaire. Eight individuals were randomly selected for a wait list, in case individuals did not show up at the specified time.

After the participants were settled, local survey staff explained the game in the local language of Kinyarwanda. Communication during the game was not permitted, except to ask questions. A significant amount of time was spent explaining the game, including the tradeoffs between private and public benefits, providing a demonstration, and conducting a full practice session. Individual decisions were completely private and anonymous; at no time were individual contributions revealed, a fact emphasized to participants.

The format of the games was standard, with a multiplier of three used to multiply the total contributions to the group. The endowment was 400 RWF, split into 4 coins of 100 RWF each.[1] There were two paid rounds, with one round of practice, using paper tickets, conducted before the paid rounds. During the paid rounds, individuals were asked to go to a completely private area, one by one, and decide how much to contribute to the group fund. To make their contribution, each individual was given a small green coin purse, with which they could deposit their preferred contribution. The money they preferred to keep for themselves was simply kept on their person. This had both the advantage of being a natural place to store money, as well as making it salient that this money was theirs to keep. Each individual amount was recorded,

*Schulich School of Business, York University, 4700 Keele Street, Toronto, Ontario, Canada M3J 1P3, and NOVAFRICA; acoutts@schulich.yorku.ca

[1] At the time, 400 RWF was approximately 0.60 USD. From the Integrated Household Living Conditions Survey 2010-2011, 400 RWF comprises of more than an average day's income for 45% of the district population. Earnings in the experiment were slightly larger than 1800 RWF on average, which greatly exceeds a day's income for the majority of the population. Real money was used ensure that the stakes were salient, and to minimize confusion.

using anonymous ID numbers located inside the contribution purses, to prevent identification of individuals by the survey team. Next, all the purses were emptied publicly one by one, and in a transparent manner the coins were counted, tripled, and divided equally among all 12 participants. Participants then received this income, and put it with the rest of their personal money.

Figure A1: Rusizi District Sectors

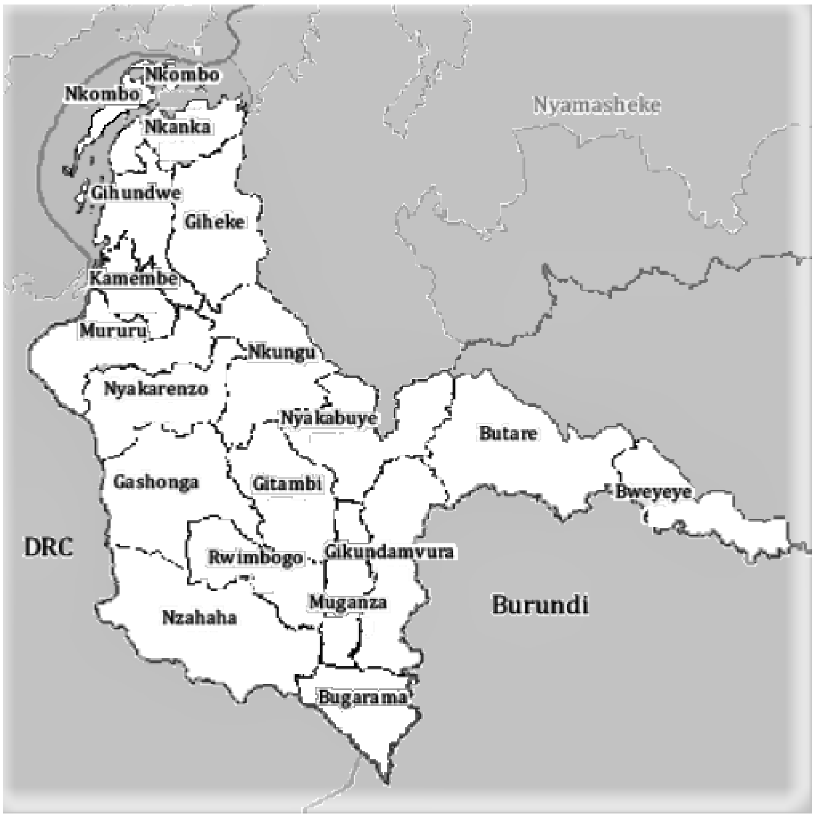

The 18 political sectors of Rusizi district. The district is bordered by both DRC and Burundi. Nyamasheke is the bordering district within Rwanda. Map is adapted from Fourth Population and Housing Census, National Institute of Statistics of Rwanda.

Subjects played two rounds of the public goods game with real stakes, receiving income directly after each round. The second round consisted of one of four different versions of the game, detailed in Section G and in the protocol below. Subjects were aware that there would be a second round, but were not given any information about the specific variation that would be used, ensuring comparability in the first round across villages. For this reason, only the first round is used for the primary analysis of the paper, while the second round is considered when exploring differential effects of spillovers on cooperation across these versions, as well as when examining potential mechanisms.

Additional data notes:

- In three villages it was not possible to find 12 participants. These are excluded from the data.
- 14 individuals are missing from the final data. Their respective village averages have been calculated excluding their data.

- One village is missing data for number of households: this value has been imputed using the observed relationship between number of households and number of households interviewed as part of the community health project's baseline survey.

A.2 Summary Statistics

Figure A2 presents the distribution of contributions in the public goods games. The possible levels ranged from 0 to 400 RWF, in 100 RWF increments. The average level contributed to the group fund is 255 RWF, which is about 64% of the socially optimal level of contributing the maximum 400 RWF.

Table A1 presents summary statistics of these village level contributions in the games, as well as variables which were collected prior to participation in the games. Women were over-represented in the larger survey, and subsequently are 74% of the participants in the experimental games.[2] The average years of completed education is 4.5, which corresponds to partially completing primary school, while the average age was 35. The average number of "strong ties" is approximately 2.6.[3] The *Community Cooperation Index* and the *Community Effort Index* were responses to a survey question on whether they agreed that community members (1) cooperate with each-other, and (2) were willing to exert effort in the community. The trust index is a binary survey response to whether they believe we can trust people or not (in general).

Figure A2: Distribution of Contributions (RWF)

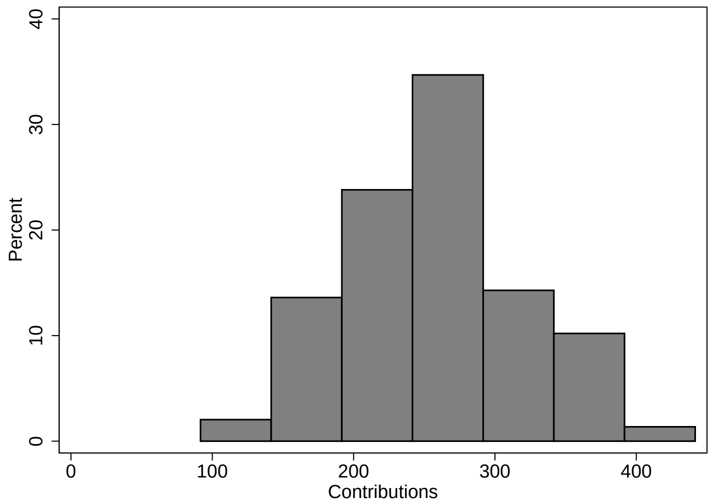

Village average contributions in the public goods experiments. Possible values ranged from 0 to 400 RWF, in 100 RWF increments. N = 147.

[2]Women were over-represented in this larger survey because the respondent was not randomly selected from within the household. Of note is that the gender ratio in Rusizi district is highly skewed, so the over-representation is not as large as it appears. According to the 2013 Rusizi District Gender Statistics Report, the percentage of females aged 25-64 is 55.4%. This age group represents 89% of those participating in this study.

[3]Strong ties refers to how many of the other 11 participants the individual knew well, by first and last name. Without this name requirement, piloting indicated that nearly all individuals reported the maximum, 11.

Table A1: Summary of Village Level Variables

	Mean	Std. Dev.	Min.	Max.
Contribution	255.38	65.23	91.7	400.0
Female	0.74	0.14	0.3	1.0
Age	35.38	3.39	26.6	45.3
Years of Education	4.47	1.28	0.8	8.5
Number of Strong Ties	2.57	1.07	0.7	6.8
Community Cooperation Index	0.82	0.13	0.4	1.0
Community Effort Index	0.79	0.16	0.3	1.0
General Trust Index	0.73	0.15	0.3	1.0
Distance to base (km)	13.14	7.98	1.0	38.8
Village Size (# HHs)	131.64	42.70	43.0	345.0
# Villages $\leq$ 1.75 km	1.86	1.33	0.0	5.0
Distance to paved road (km)	4.73	3.81	0.0	14.1
Observations	147			

Number of Strong Ties: How many others (of 11) know well, and know full legal name. *Community Cooperation Index*: Derives from whether participants agree (1), neither agree nor disagree (0.5), or disagree (0) that people in community generally cooperate. *Community Effort Index*: Derives from whether participants agree (1), neither agree nor disagree (0.5), or disagree (0) that people in community are willing to exert effort towards improving community. *General Trust Index*: Derives from whether participants agree we can generally trust people. *Distance to base*: Distance from village to study base location in kilometers. *# Villages* $\leq 1.75km$: Number of other villages within 1.75km in study; includes both villages with earlier and later participation dates. *Distance to paved road*: Distance from village to nearest paved road in kilometers.

A.3 Participant Script

Disclosure: I did not have the exact, original instructions used in the field, but have recreated them to the best of my ability here.[4]

Rusizi Evaluation of Community Hygiene Clubs
Participant Script
Voluntary Contribution Exercises

[These instructions should be read out to the participants before commencing of the activity. Each group will only complete one of the four possible versions of Round 2]

Enumerator instructions: Ensure that the area is clear of spectators, and that each participant has been given the correctly numbered green purse.

Activity Description

Thank you for agreeing to participate in this activity. In this activity, you will be making decisions that can earn you money. The money you earn depends on your choices, as well as the choices of others. We will play the activity two times. Now we will explain the rules for the first time. The second time may be slightly different, and it will be explained when we get there.

[4] Instructions were delivered in Kinyarwanda.

Don't worry, we will answer any questions and conduct a practice before we begin.

As you can see, there are twelve of you in this room, and each of you has been given a green coin purse. Everyone will equally be given four 100 RWF coins (a total of 400 RWF).

The objective of the activity is for you to use the money provided in one of two ways. One option is to contribute some, or all of it, towards a group fund, by putting it in the green coin purse. The other option is to keep some, or all of it, for yourself. Regardless of what you do, this decision is private, and no-one will know what decision you choose.

All money contributed to the group fund will be added together, tripled, and then evenly split amongst all 12 participants, independent of their contribution.

Any money that you keep for yourself is directly yours to keep. Regardless of how much money you decide to keep for yourself, you will still share equally in the group fund.

Here is how you will make your choice of how much money (of the 400 RWF) to contribute to the group fund or keep for yourself. One by one, you will be asked to come to a private area, where no one can see your decisions. In this area you will allocate the money as you like. The money you choose to contribute to the group fund, you will place in the green coin purse. The money you will keep your yourself, you will keep on your person.

After making your allocation, you will bring your green coin purse to the "banker". After all 12 of you have dropped off your green purses, the banker will count all of the coins in all of the green purses, multiple them by three, and put an equal number of coins in every coin purse, and we will return them to you.

Before we start we will show an example.

Enumerator instructions: Go through the three examples with four mock participants. Example 1: Everyone contributes 0 to group, and everyone receives 400. Example 2: Everyone contributes 400 to group, and everyone receives 1200. Example 3: Three participants contribute 400, one contributes 0. The first three participants receive 900, the fourth receives 1300.

Are there any questions?

Now we will perform one practice round. Instead of 4 coins, now you will receive 4 tickets. Remember this is just for practice and not for money. Remember to respect the privacy of everyone and ensure there is no talking during the decisions.

Conduct full practice round.

Are there any questions? Remember to respect the privacy of everyone and ensure there is no talking during the decisions.

ROUND 1: BASELINE:

Enumerator instructions: One by one ask the participants to go to the private area to make their decisions. Participants will drop off green coin purses with the banker.

After all purses have been dropped off, the banker will count all the coins in front of all participants. Then the banker will triple (adding two coins for every one) the amount and return an equal amount back to every coin purse. Next each participant will receive their coin purse back and can put their earned money aside.

Note: Only one of the following four versions will be played:

ROUND 2: BASELINE (AGAIN):

Now we will play the second and final round of the activity. The second round is identical to the previous one. So we will now proceed.

Enumerator instructions: One by one ask the participants to go to the private area to make their decisions. Participants will drop off green coin purses with the banker.

After all purses have been dropped off, the banker will count all the coins in front of all participants. Then the banker will triple (adding two coins for every one) the amount and return an equal amount back to every coin purse. Next each participant will receive their coin purse back and can put their earned money aside.

ROUND 2: PENALTY:

Now we will play the second and final round of the activity. The second round is similar to the previous one. The only difference is that now, after everyone has made their contribution decisions, you will receive the green coin purse of another randomly chosen person in this room. This also means that someone else will have your green coin purse.

Without knowing who this person is, you will be able to see how many coins they contributed to the group fund. If you want, you will have the option to give this person a penalty. If you give them a penalty, the banker will remove 100 RWF from their earnings. But, giving a penalty will also cost you 50 RWF, which the banker will take from your earnings.

Remember, that someone will also see how much you contributed, and can choose to give you a penalty.

Before we begin, each of you will be given a ticket marked with an 'X'. To give a penalty to the person whose purse you hold, all you have to do is put this ticket inside the purse. If you do not want to give a penalty, keep the ticket, and do nothing.

Are there any questions?

Enumerator instructions: One by one ask the participants to go to the private area to make their decisions. Participants will drop off green coin purses with the banker.

After all purses have been dropped off, the banker will distribute these coin purses according to a pre-randomized order. Each participant will examine how many coins are inside the purse, and decide whether or not to give a penalty, by placing the ticket inside.

Next the coin purses are returned to the banker who will count the coins in front of all participants. Then the banker will triple (adding two coins for every one) the amount and return an equal amount back to every coin purse. The banker will remove 50 RWF for those who gave a penalty, and 100 RWF for those who received a penalty. Next each participant will receive their coin purse back and can put their earned money aside.

ROUND 2: REWARD:

Now we will play the second and final round of the activity. The second round is similar to the previous one. The only difference is that now, after everyone has made their contribution decisions, you will receive the green coin purse of another randomly chosen person in this room. This also means that someone else will have your green coin purse.

Without knowing who this person is, you will be able to see how many coins they contributed to the group fund. If you want, you will have the option to give this person a reward. If you give them a reward, the banker will add 100 RWF from their earnings. But, giving a reward will cost you 50 RWF, which the banker will take from your earnings.

Remember, that someone will also see how much you contributed, and can choose to give you a reward.

Before we begin, each of you will be given a ticket marked with an 'X'. To give a reward to the person whose purse you hold, all you have to do is put this ticket inside the purse. If you do not want to give a reward, keep the ticket, and do nothing.

Are there any questions?

Enumerator instructions: One by one ask the participants to go to the private area to make their decisions. Participants will drop off green coin purses with the banker.

After all purses have been dropped off, the banker will distribute these coin purses according to a pre-randomized order. Each participant will examine how many coins are inside the purse, and decide whether or not to give a reward, by placing the ticket inside.

Next the coin purses are returned to the banker who will count the coins in front of all participants. Then the banker will triple (adding two coins for every one) the amount and return an equal amount back to every coin purse. The banker will remove 50 RWF for those who gave a reward, and add 100 RWF for those who received a reward. Next each participant will receive their coin purse back and can put their earned money aside.

ROUND 2: UNCERTAINTY

Now we will play the second and final round of the activity. The second round is similar to the previous one. The only difference is that now, each of you will be given an additional blue coin purse. You will make your contributions as before, but this time there are two group funds, the green purse fund and the blue purse fund.

You can decide to contribute however you like – but money in one of these funds will be multiplied by 5. In the other fund the money will not change at all. Importantly, which fund is multiplied by 5 will be determined at random, by a coin toss, independent of your decisions. Therefore, you will not know in advance which fund is multiplied by 5, and which fund will stay the same.

The rest of the activity will be similar. After making all of your decisions in private, the banker will flip a coin to determine which fund is multiplied by 5. After this, the banker will count all of the coins in the green and blue purses, separately. Then, the selected funds coins will be multiplied by 5. The banker will add all the coins together, and divide them equally among all participants, exactly as before.

Are there any questions?

Enumerator instructions: One by one ask the participants to go to the private area to make their decisions. Participants will drop off green and blue coin purses with the banker.

After all purses have been dropped off, the banker will flip a coin in front of everyone. The outcome of the flip will determine which fund is multiplied by 5.

Next the banker will count the coins for each fund in front of all participants. Then the banker will multiply by 5 (adding four coins for every one) for the fund that was selected by the coin toss. The other fund will remain with the same number of coins. Next the banker will total all of the coins and return an equal amount back to every coin purse. Next each participant will receive their coin purse back and can put their earned money aside.

B Determinants of Visit Order

Table B1 presents an estimation of the order of visits, conditional on the variables observed by the planner, including the sectors. This regression shows that with an R^2 of 0.93, these variables are sufficient to explain 93% of the variation in the order of visits.

Table B1: Determinants of Visit Order

	(1)
Distance to base (km)	0.433^{**}
	(0.215)
Distance to paved road (km)	0.225
	(0.202)
Village Size (# HHs)	0.006
	(0.006)
# Villages $\leq$ 1.75 km	-0.177
	(0.213)
Sector Fixed Effects	✓
R^2	0.93
Observations	147

Analysis uses OLS regression. Significantly different from 0 at * 0.1; ** 0.05; *** 0.01. Robust standard errors in parentheses. Dependent variable indicates order of visit and varies from 1 to 53. Villages visited on the same day receive the same value of this variable.

Figure B1 shows the histogram of the residuals from the regression in Table B1. The figure shows there are still deviations from predicted visit order, as 59% of villages are outside the interval $[-1, 1]$, indicated by the red lines.

Figure B1: Deviations from Predicted Order

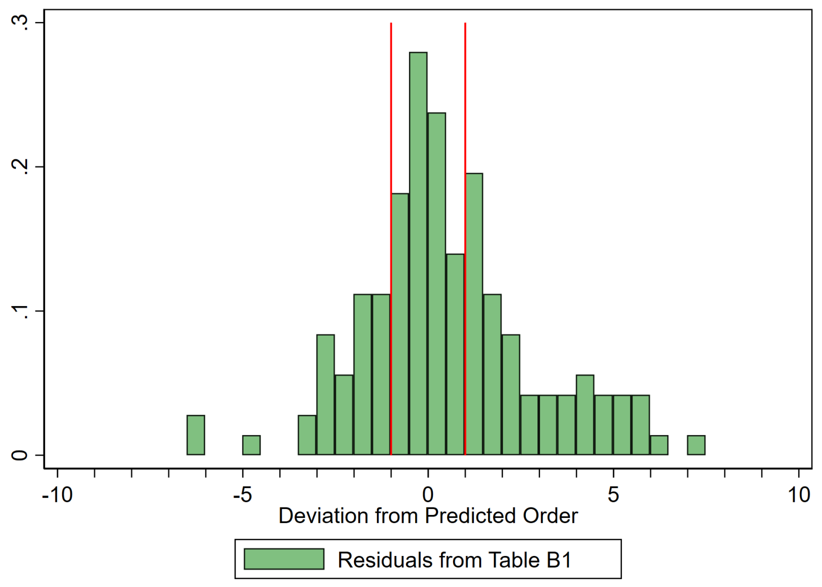

Histogram of residuals from Table B1. $N = 143$ villages; four outliers ($|res_i| > 10$) excluded.

C Matching Strategy

C.1 Pre-Matching Statistics for Main Treatment

Table C1 presents a logit regression of village characteristics on the main treatment (1.75 km cut-off) indicator for potential communication. Among the variables that the planner had access to, only village density is significant. Note that a small proportion of villages (16%), had no neighbors within 1.75 km. These are dropped from the matching strategy.[5] As noted in the main paper, exact matching is also performed on village density, in Section D, alleviating specific concerns with this imbalance. As an additional check there is no statistically significant relationship between contributions in the public goods games and village density.[6]

Table C1: Logit regression for treatment: Past participating neighbors within 1.75 km

**Logit Regression**	**Treatment**
	(1)
Distance to base (km)	0.034
	(0.109)
Village Size (# HHs)	-0.005
	(0.006)
# Villages $\leq$ 1.75 km	1.093***
	(0.221)
Distance to paved road (km)	0.050
	(0.153)
Observations	144
Sector Fixed Effects	✓

Note: *** p<0.01, ** p<0.05, * p<0.1. Robust standard errors are reported in parenthesis. One sector is dropped (3 villages) due to lack of variation in treatment status.

Next Table C2 examines the balance across treated and control villages. Regarding variables available to the planner, again village density is significant, while distances to the study's base location or to the nearest paved road are not significantly different across the groups, nor is the village size. Turning to the variables unavailable to the planner, the only statistically significant variable is average contributions: contributions are 30 RWF greater in treatment villages. Attitudes towards cooperation (measured before participation) are not significantly different.

As an additional check of balance, Table C3 presents additional variables coming from the baseline survey of the evaluation of community health clubs, which was conducted just before the public goods games. One village was not able to be matched. Due to the nature of that evaluation, many of the variables are focused on health, while there are a few which relate to household assets and construction quality. There were no questions on religion nor on ethnicity (not permitted by Rwandan law). From Table C3 one can see that from this small set of variables, there are no significant differences across the villages assigned to treatment versus control.

[5] If one includes these villages, 88% of them end up being removed anyway due to extremely low propensity score values that fall outside of the common support. As such, including them does not alter the main results.

[6] They are positively correlated. The p-value corresponding to an F-test for whether village density can explain contributions is 0.116. Adding the treatment dummy reduces this p-value to 0.763. Additionally village density is uncorrelated with contributions in the control group (F-test p-value 0.812). This relationship is investigated further using OLS regressions in Online Appendix Tables E7 and E8. Finally, an additional reason this may be less of an issue than first appearances suggest is that the variable is defined as neighboring villages *in the study*. Since only 150 villages out of 598 participated in the study, the variable itself is only correlated with the actual number of neighbors.

Table C2: Balance of main treatment: Past participating neighbors within 1.75 km

	Treatment	Control	Difference
Available to planner			
Distance to base (km)	12.18	14.09	-1.91
Village Size (# HHs)	128.60	134.64	-6.04
# Villages $\leq$ 1.75 km	2.48	1.24	1.24***
Distance to paved road (km)	4.96	4.50	0.45
Unavailable to planner			
Contribution	270.61	240.36	30.25***
Female	0.75	0.73	0.01
Age	35.37	35.39	-0.02
Years of Education	4.55	4.38	0.17
Community Cooperation Index	0.81	0.82	-0.01
Community Effort Index	0.79	0.79	-0.00
Number of Strong Ties	2.54	2.59	-0.04
General Trust Index	0.72	0.74	-0.02
Observations	73	74	147

Significant differences indicated by * 0.1; ** 0.05; *** 0.01.

Table C3: Balance of main treatment (Supplemental Variables)

	Treatment	Control	Difference
Unavailable to planner			
Anyone with diarrhea $<$ 7 days	0.17	0.18	-0.01
Anyone ill or injured $<$ 4 weeks	0.60	0.60	-0.00
Number of livestock	2.34	2.46	-0.11
Durable asset index	0.77	0.73	0.04
Anyone didn't sleep under bed net	0.12	0.14	-0.02
Log of value of house	12.97	12.84	0.13
Housing materials index	0.67	0.65	0.02
Anyone take medication for fever $<$ 4 weeks	0.26	0.27	-0.01
Observations	73	73	146

Significant differences indicated by * 0.1; ** 0.05; *** 0.01.

C.2 Propensity Score Matching: Theory and Implementation

Here I follow the notation of Imbens (2015), with some slight adaptations. Let $C_i(1)$ denote the outcome of interest, village level contributions, if village i had at least one neighboring village within 1.75 km which previously participated in the games (treated, $W_i = 1$), and $C_i(0)$ be the contribution of a village with no previous neighbours (untreated, $W_i = 0$). In an ideal world, one could observe both outcomes (treated and untreated) for the same village, and hence could calculate the average treatment effect τ. The classic problem is that one cannot obtain an unbiased estimate of the treatment effect by naive comparison of the average outcomes of the two groups ($\bar{\tau} = \bar{C}(1) - \bar{C}(0)$) because these groups may have different characteristics.

In practice, randomization can solve this problem, by creating comparable treatment and control groups. Here, randomization did not occur. Instead, following Rosenbaum and Rubin (1983) and a number of others, the strategy is to find a set of observable covariates X, which are known to be not affected by the treatment, such that:

$$W_i \perp C_i(1), C_i(0)|X_i. \tag{C1}$$

This assumption is referred to as unconfoundedness or selection on observables. It means that the outcomes are uncorrelated with treatment, conditional on covariates X_i. In the current context this assumption is likely to be satisfied. The reason is that the treatment W_i (being exposed to villages who previously participated) could only have been conditioned on observables. This is because, as stated in the paper, the planner determined the order of visits, in advance, with a limited number of pre-visit observables, and had no prior familiarity with the villages. In particular, it would be impossible for the planner to condition the treatment on features of data which had not yet been collected.

Denote the propensity score, $e(x)$ by:

$$e(x) = Pr(W_i = 1|X_i = x), \tag{C2}$$

i.e. the probability that a village receives the treatment conditional on having characteristics $X_i = x$. This is also equivalent to the expectation of the treatment, $\mathbb{E}[W_i = 1|X_i = x]$.

We can thus define the average treatment effect as:

$$\tau = \mathbb{E}[\mathbb{E}[C_i|W_i = 1, X_i] - \mathbb{E}[C_i|W_i = 0|X_i]] \tag{C3}$$

As Imbens (2015) notes, in addition to unconfoundedness, a second key assumption is required for the analysis. This involves a requirement that there is overlap in the distribution of covariates across treatment and control villages. Intuitively speaking, one needs to be able to find similar villages in control and treatment groups, in order to make valid comparisons.

Regarding this second assumption of overlap, as noted earlier, in Table C2, average characteristics on variables observed by the planner are reasonably balanced across treatment and control villages. As the next section will show in more detail, the assumption of overlap is broadly supported in the data.

The propensity score needs to be estimated from the covariates which may potentially have had an impact on which villages received the treatment (having neighbors that previously participated). In the case of this study, these variables can only come from the set of all observables available to the planner at the time the order of visits was determined. In determining the propensity score, I do not include sector dummies, as sectors will be conditioned on using the exact matching strategy.

Regarding the key variables available to the planner, outlined in Section 2.3 in the main paper, one cannot assume that the planner used these variables in a linear way. Thus it is also important to take into account potential higher order interactions between these variables and the treatment. To determine the optimal specification, I follow the algorithm outlined in Imbens (2015), which involves selection of these higher order terms based on their added value in terms of predicting treatment assignment. The algorithm involves step-wise regression estimation of the propensity score, to select only those covariates that add value in determining treatment status.[7]

In fact, the algorithm does not select any additional higher order terms. Thus the final

[7] I follow Imbens (2015), and set the threshold value for second order terms to be $C_{qua} = 2.71$.

terms selected for estimation of the propensity score are solely the four variables corresponding to distance from base, distance from paved road, number of households in village, and village density within 1.75 km.[8]

Figure C1 presents the distribution of the propensity score by main treatment status. There is significant overlap over the sample, with the exception of values close to 1.[9] In the analysis I impose restrictions that matching must occur in regions with common support.

Figure C1: Propensity Scores by Main Treatment Status

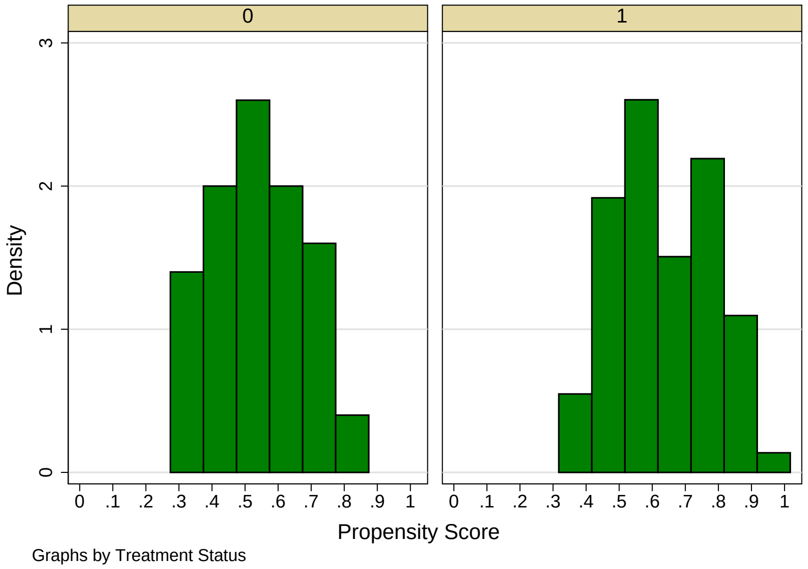

Distribution of propensity score, for main treatment (1.75 km cut-off). $N = 123$.

C.3 Matching Estimates for All Distance Cut-offs

Table C4 presents average treatment effect (ATE) estimates analogous to Figures 2a and 2b in the main paper, corresponding to standard matching and exact matching on sector respectively. Hence Table C4 contains the results of 18 different matching analyses.

[8] An important note regarding the matching analyses for other distances (which ranged from 1 km to 3km, in 0.25 km increments), is that the algorithm does select some higher order terms for the distances of 2.5 km, 2.75 km, and 3 km.

[9] If one were to include villages with no neighbors, this would also present additional imbalances in the left tail.

Table C4: Average Treatment Effect Matching Estimates

	Standard Matching			Exact Matching		
Cut-off	ATE	SE	N	ATE	SE	N
1.00 km	33.77	(30.74)	31	60.83	(69.68)	17
1.25 km	22.15	(13.51)	71	44.33*	(23.41)	49
1.50 km	15.71	(16.80)	86	32.40*	(16.70)	64
1.75 km	27.92***	(9.95)	118	36.05***	(10.93)	102
2.00 km	36.14***	(11.25)	113	36.18***	(14.82)	89
2.25 km	18.03	(12.71)	121	34.84**	(17.22)	88
2.50 km	12.41	(14.89)	114	18.94	(18.54)	82
2.75 km	6.80	(11.59)	123	21.78*	(12.75)	71
3.00 km	13.39	(14.31)	118	7.57	(14.60)	67

Analyses use nearest neighbor propensity score matching, with 2 neighbors, with replacement. Significantly different from zero at * 0.1; ** 0.05; *** 0.01. Abadie-Imbens Robust Standard Errors in parentheses. Values of propensity score outside common support range are dropped. Exact matching excludes sectors with only 0 or 1 village in either treatment or control groups.

C.4 Post-Matching Balance and Alternative Estimates for Main Treatment

Table C5 presents alternative matching estimates for the main treatment using either 1 or 3 neighbors, as opposed to 2 neighbors used in the main paper. The results vary only slightly, and are significant at the 1% level for all cases except for standard matching with 1 neighbor, where significance is at the 5% level.

Figure C2 shows the distributions of matching variables by main treatment status (1.75 km cut-off), in order to evaluate balance of the matching strategy. There are not large imbalances across these variables, even in the unmatched stage. Matching improves balance, which is slightly better in standard matching (column a) rather than exact matching (column b), though the differences are not substantial.

Table C5: Matching Estimates (Main Treatment): Alternative # Neighbors

	(1)<br>Standard Matching	(2)<br>Exact Matching
	1 Neighbor	
ATE	24.845**<br>(10.189)	32.033***<br>(10.614)
	3 Neighbors	
ATE	31.591***<br>(9.822)	30.335***<br>(10.452)
Observations	118	102

Analysis uses nearest neighbor propensity score matching, with 1 or 3 neighbors, with replacement. Significantly different from zero at * 0.1; ** 0.05; *** 0.01. Abadie-Imbens Robust Standard Errors in parentheses. Values of propensity score outside common support range are dropped. Exact matching excludes sectors with only 0 or 1 village in either treatment or control groups.

Figure C2: Balance of Matching Variables (Main Treatment)

(a) Standard Matching | (b) Exact Matching

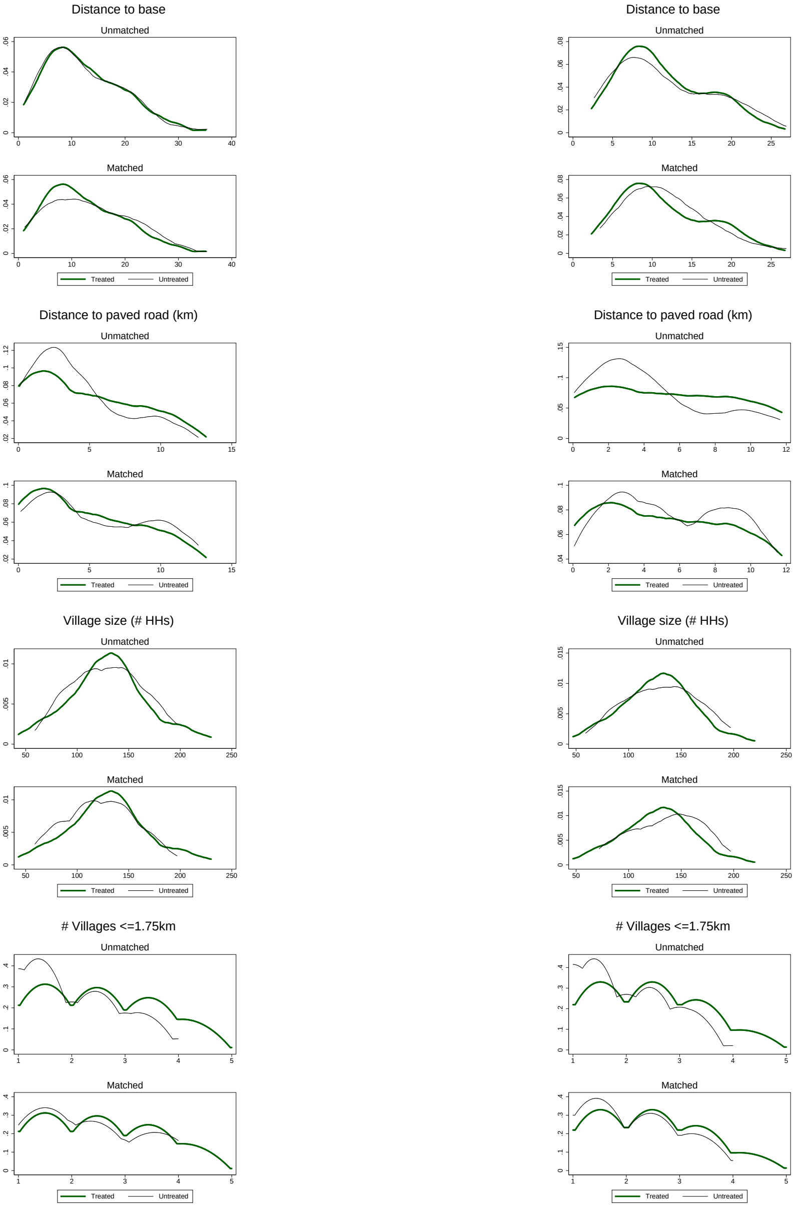

Density of covariates in matching estimations by main treatment status (1.75 km cut-off), before and after matching. **(a)** $N = 118$ **(b)** $N = 102$.

D Exact Matching on Village Density (Main Treatment)

Here I address concerns that treatment effects may be picking up differences in village densities, i.e. the number of neighboring villages in the study located within 1.75 km.[10] By construction, the treatment variable is correlated with the number of total neighbors within 1.75 km in the study. Further, the initial balance checks showed an imbalance in village density across treatment and control villages.

To account for the possibility that village density may be correlated with unobserved variables, and that the earlier propensity score matching may have been unable to adequately control for this, I force exact matching on number of neighbors in the study, village density. Village density has a minimum value of 0 and a maximum of 5, though values of 0 are excluded given they can never be treated. Additionally, as there are no control villages for densities with 5 neighbors, these villages are necessarily dropped from this analysis as well. Table D1 presents this matching analysis. The estimated effects are significant at the 1% level, and consistent with the main results.

Table D1: Average Effect of Presence of Past Participating Neighbors

	Exact Matching on Number Villages within 1.75 km
ATE	31.752***
	(11.089)
Observations	119

Analysis uses nearest neighbor propensity score matching, with 2 neighbors, with replacement. Significantly different from zero at * 0.1; ** 0.05; *** 0.01. Abadie-Imbens Robust Standard Errors in parentheses. Values of propensity score outside common support range are dropped. Exact matching excludes densities with only 0 or 1 village in either treatment or control groups.

E OLS Specifications, Robustness, and Placebo Checks

E.1 OLS Specifications Corresponding to Matching Analysis

It is also useful to examine OLS specifications, particularly as the matching strategy substantially reduces the number of observations. The validity of the unconfoundedness assumption that underlies the matching strategy is also required for unbiased OLS estimation. Beyond this, OLS requires an assumption of linearity of the conditional expectations of outcomes, conditional on the covariates (Imbens and Wooldridge, 2009).

Table E1 first presents a simple OLS specification of the impact of having neighboring villages within 1.75 km who previously participated (i.e., the main treatment), on contributions. It is possible to see that the impact is positive and significant, consistent with the matching estimates. The corresponding coefficient in the specification with all controls in column 3 is 27.7 RWF, an 11% increase from average contributions, and significant at the 5% level. Of note is that the estimate varies only slightly when additional controls are added, including sector fixed effects. This is consistent with evidence from Table C2 which demonstrated that the treatment was not significantly correlated with most observables. Beyond this, it is reassuring that the only variable significantly associated with treatment, the

[10]Note that matching on density is not well suited for large or small distance cut-offs given the resulting imbalances across treatment and control. Unlike exact matching on sector, density is correlated with treatment status.

number of neighboring villages within 1.75 km, is not significant in the regression. Notably, the results obtained are quantitatively similar to those obtained using matching.

Another advantage of the OLS specification is that it showcases the relationship between contributions and other observables of interest. Table E1 shows that female participants give significantly more than others. Perhaps it is surprising that trust, cooperation, and community effort are not significant. However, this is partially an artifact of the high correlation among these variables (their pairwise correlations vary from 0.48 to 0.58). If these variables were to enter individually, the trust and cooperation measures are positive and significant at the 5% and 10% level respectively, in column 3. Another surprising result is that the number of strong ties is significantly associated with fewer contributions. I do not have an explanation for this result, although it should be noted that *strong ties* indicates the number of others one knows by both first and last name. Thus these links can be unilateral, and may not be social in nature.

Finally, it is useful to showcase the OLS specifications for all distance cut-offs from 1 km to 3 km, corresponding to Figures 2a and 2b in the main paper. Figure E1 presents these estimates, using the specification with full controls analogous to column 3 in Table E1. These results show a pattern of diminishing effects as the distance cut-off is greater. The largest effects are found for villages with neighbors within 1km, increasing contributions by 49.0 significant at the 1% level, and the smallest at 2.75 km, increasing contributions by 9.9 RWF, not significant at conventional levels. As in the main paper, the main treatment is highlighted in purple with a diamond marker.

Figure E1: OLS estimates for different treatment cut-off distances

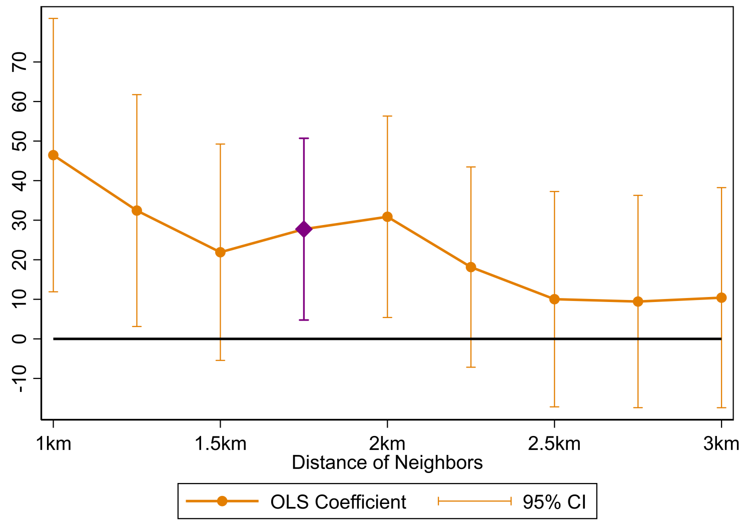

Each point corresponds to estimate of coefficient on treatment for specified distance cut-off for independent OLS regressions, analogous to column 3 of Table E1. N = 147.

Table E1: Effect of Presence of Past Participating Neighbors Within 1.75 km

	(1)	(2)	(3)
Treatment Status	30.247***	28.375***	27.736**
	(10.517)	(10.214)	(11.623)
Distance to base (km)		−0.072	−1.001
		(0.830)	(3.182)
Distance to paved road (km)		4.354**	0.973
		(1.705)	(4.435)
Village Size (# HHs)		0.010	0.090
		(0.112)	(0.138)
# Villages $\leq$ 1.75 km		0.243	2.665
		(4.711)	(5.612)
Years of Education		2.312	−0.027
		(4.905)	(6.347)
Female		97.033***	86.295**
		(37.274)	(43.021)
Age		12.839	16.726
		(26.332)	(25.826)
Age^2		−0.171	−0.234
		(0.364)	(0.359)
General Trust Index		63.993	75.653
		(48.118)	(48.621)
Community Cooperation Index		53.488	55.765
		(51.655)	(65.730)
Community Effort Index		−20.366	−26.792
		(40.272)	(41.970)
Number of Strong Ties		−11.913**	−9.792*
		(5.420)	(5.859)
Sector Fixed Effects			✓
R^2	0.05	0.20	0.30
Observations	147	147	147

Analysis uses OLS regression. Dependent variable is contributions. Significantly different from 0 at * 0.1; ** 0.05; *** 0.01. Robust standard errors in parentheses.

E.2 Alternative Definition of Village Density

The OLS specifications above require that conditional on village density (# Villages $\leq$ 1.75 km), treatment is random. This condition is reminiscent of the methodology of papers such as Kremer and Miguel (2007) and Oster and Thornton (2012). Here, a non-linear relationship between village density and treatment could bias the OLS estimates. Focusing on the main treatment specification shown in Table E1, the probability of treatment is indeed not a linear function of village density: the change in the probability of being treated increases more as one moves from 0 to 1 neighbors, than if one moves from 4 to 5 neighbors.

To account for this, Table E2 replaces the village density variable with the conditional probability of treatment, for each village density value from 0 to 5. These probabilities are respectively: 0, 0.44, 0.61, 0.63, 0.82, and 1. From Table E2 one can see that the estimates of treatment status are not substantively affected by this modification, indicating that in the current context the non-linearity is not a cause for concern. I thank an anonymous referee for

bringing this concern and its solution to my attention.

Table E2: Effect of Presence of Past Participating Neighbors Within 1.75 km

	(1)	(2)	(3)
Treatment Status	30.247^{***}	32.613^{***}	30.464^{**}
	(10.517)	(10.620)	(11.874)
Distance to base (km)		-0.183	-1.059
		(0.826)	(3.209)
Distance to paved road (km)		4.377^{***}	1.260
		(1.700)	(4.463)
Village Size (# HHs)		0.013	0.090
		(0.111)	(0.137)
Prob. Treatment		-16.600	-0.698
		(23.750)	(25.654)
Years of Education		2.705	0.307
		(4.902)	(6.309)
Female		101.501^{***}	89.976^{**}
		(36.870)	(43.385)
Controls		✓	✓
Sector Fixed Effects			✓
R^2	0.05	0.20	0.29
Observations	147	147	147

Analysis uses OLS regression. Dependent variable is contributions. Significantly different from 0 at * 0.1; ** 0.05; *** 0.01. Robust standard errors in parentheses. Controls includes all remaining variables found in Table E1. Prob. Treatment is equal to the empirical probability of treatment, conditional on the value of village density (# villages within 1.75 km).

E.3 OLS Robustness I: Alternative Specifications

In this section I examine three alternative variables to the defined treatment which are likely to be correlated with opportunities for communication with past participants, and thus expected to have a positive effect on cooperation. In the following Section E.4, I examine two placebo tests with variables which relate to the overall number of villages (either in the sector or in the 1.75 km radius), and show that these are not significantly related to contributions.

Table E3 presents the effect of the order that a village was visited within a sector. That is, a village that is the very first to participate in the public goods games in its sector would be coded as 1, the village that is the second to be visited within its sector would be coded as 2, and so on. Then the variable is standardized to be mean zero, standard deviation one, in order to facilitate comparison across tables in this section.

Villages that participate first in their sector will be very unlikely to have had contact with previous participants. While villages who participate after 5 villages in their sector had participated, will be much more likely to have had such contact. Consistent with this and the results of the main paper, Table E3 shows that the order of visit within sector is associated with significantly higher contributions.

The next Table E4 simply counts the number of villages within the sector which previously participated in the games, then standardizes it. Again this variable is positive and significant in

determining contributions.[11]

Finally Table E5 examines the effect of a variable defined as the distance to the nearest past participating village. This variable ranges from 0.2 km to 27 km, with a mean value of 2.5 km. As very few villages have distant neighbors, this variable is top-coded at the 95th percentile, 5.17km.[12] From column 3, one can see that for every additional kilometer to the nearest participating village, contributions are reduced by 9.2, or about 4% of the average.

Table E3: Effect of Ranking of Order Visited Within Sector

	(1)	(2)	(3)
Rank of Visit (within Sector)	16.423^{***}	15.339^{***}	17.291^{***}
	(5.145)	(4.688)	(6.043)
Distance to base (km)		0.214	-1.670
		(0.863)	(2.982)
Distance to paved road (km)		3.810^{**}	-0.303
		(1.750)	(4.382)
Village Size (# HHs)		0.005	0.062
		(0.112)	(0.132)
# Villages $\leq$ 1.75 km		5.345	8.828^{*}
		(4.481)	(4.902)
Years of Education		3.481	3.182
		(4.738)	(6.607)
Female		104.160^{***}	90.462^{**}
		(35.737)	(40.331)
Controls		✓	✓
Sector Fixed Effects			✓
R^2	0.06	0.21	0.31
Observations	147	147	147

Analysis uses OLS regression. Dependent variable is contributions. Significantly different from 0 at * 0.1; ** 0.05; *** 0.01. Robust standard errors in parentheses. Variable "Rank of Visit (within Sector)" has been standardized. Controls includes all remaining variables found in Table E1.

[11]As a placebo test, in the next section, Table E6 shows that the total number of villages in the sector is not driving this result.

[12]The reason for top-coding is that one would not expect any relationship between distance and cooperation among the further distances, since these villages are most likely to have had no communication independent of distance. Hence these distances simply introduce noise. Without top-coding, the estimates continue to be significant in column 3, but not in columns 1 and 2.

Table E4: Effect of Number of Previous Participating Villages in Sector

	(1)	(2)	(3)
# Villages before in Sector	12.987***	13.938***	15.185**
	(4.953)	(4.886)	(5.911)
Distance to base (km)		0.437	−1.762
		(0.858)	(2.997)
Distance to paved road (km)		4.600***	−0.052
		(1.772)	(4.390)
Village Size (# HHs)		−0.007	0.055
		(0.111)	(0.133)
# Villages $\leq$ 1.75 km		5.991	9.413*
		(4.478)	(4.851)
Years of Education		4.717	2.843
		(4.854)	(6.471)
Female		101.796***	88.682**
		(36.401)	(40.652)
Controls		✓	✓
Sector Fixed Effects			✓
R^2	0.04	0.21	0.30
Observations	147	147	147

Analysis uses OLS regression. Dependent variable is contributions. Significantly different from 0 at * 0.1; ** 0.05; *** 0.01. Robust standard errors in parentheses. Variable "# Villages before in Sector" has been standardized. Controls includes all remaining variables found in Table E1.

Table E5: Effect of Distance to Nearest Past Participating Village

	(1)	(2)	(3)
Distance to Nearest Past Participant	−8.939**	−7.477**	−9.166**
	(3.724)	(3.743)	(4.252)
Distance to base (km)		0.049	−0.659
		(0.837)	(3.208)
Distance to paved road (km)		4.573**	1.787
		(1.789)	(4.872)
Village Size (# HHs)		−0.034	0.039
		(0.116)	(0.143)
# Villages $\leq$ 1.75 km		3.147	6.028
		(4.437)	(5.031)
Years of Education		4.237	1.201
		(5.129)	(6.681)
Female		89.284**	80.349*
		(40.385)	(43.581)
Controls		✓	✓
Sector Fixed Effects			✓
R^2	0.03	0.17	0.27
Observations	144	144	144

Analysis uses OLS regression. Dependent variable is contributions. Significantly different from 0 at * 0.1; ** 0.05; *** 0.01. Robust standard errors in parentheses. Controls includes all remaining variables found in Table E1. Distance to nearest past participant is in kilometers, and is top-coded at the 95th percentile (5.17km). The 3 missing observations are because the first 3 villages (on day 1) have no past participating neighbors.

E.4 OLS Robustness II: Placebo Specifications

This section presents three placebo tests for measuring opportunities for communication. The previous Table E4 examined the number of villages that were previously visited within the same sector, and found a strong significant relation with contributions. Table E6 examines the total number of villages in the sector, irrespective of the date of visit. This is correlated, but not significantly associated with contributions. Note that this variable is standardized to have mean zero, standard deviation one, to be comparable with the other tables in this section.

The second and third placebo tests are in fact the variable reflecting village density, the number of villages within 1.75 km, irrespective of the sector or date of visit. This variable is a key matching variable, but it is of note that even though it is correlated with treatment, there are not statistically significant effects of village density on contributions, shown in Table E7. A positive coefficient is to be expected, given that the variable is correlated with treatment. It is significantly diminished when the treatment variable is added to the regression (column 4). Again note that the variable has been standardized.

Table E8 presents this same analysis, but restricted only to the control sample. That is, these regressions examine the effect of the total number of neighboring villages within 1.75km, for the case when none of these neighboring villages participated before. Reassuringly, there is no relationship between village density and contributions. Additionally the coefficient is much smaller in magnitude than Table E7, and sometimes negative.

Table E6: Effect of Total Number of Villages Within Sector

	(1)	(2)
# Villages in Sector	5.869	4.973
	(4.620)	(5.126)
Distance to base (km)		0.216
		(0.913)
Distance to paved road (km)		4.720***
		(1.806)
Village Size (# HHs)		−0.002
		(0.117)
# Villages $\leq$ 1.75 km		5.284
		(4.538)
Years of Education		3.257
		(4.973)
Female		92.697**
		(38.471)
Controls		✓
Sector Fixed Effects		
R^2	0.01	0.17
Observations	147	147

Analysis uses OLS regression. Dependent variable is contributions. Significantly different from 0 at * 0.1; ** 0.05; *** 0.01. Robust standard errors in parentheses. Sector fixed effects omitted as there is no variation across sectors in number of villages within the sector. Variable has been standardized. Controls includes all remaining variables found in Table Controls includes all remaining variables found in Table E1.

Table E7: Effect of Number of Neighbors Within 1.75 km

	(1)	(2)	(3)	(4)
# Villages $\leq$ 1.75 km	8.857	7.567	11.182	3.696
	(5.874)	(6.283)	(6.866)	(7.783)
Treatment				27.736**
				(11.623)
Distance to base (km)		−0.087	−0.835	−1.001
		(0.860)	(3.163)	(3.182)
Distance to paved road (km)		4.782***	0.848	0.973
		(1.811)	(4.424)	(4.435)
Village Size (# HHs)		−0.012	0.061	0.090
		(0.120)	(0.139)	(0.138)
Years of Education		2.653	0.121	−0.027
		(4.919)	(6.299)	(6.347)
Female		93.179**	74.891*	86.295**
		(38.157)	(40.966)	(43.021)
Controls		✓	✓	✓
Sector Fixed Effects			✓	✓
R^2	0.02	0.17	0.27	0.30
Observations	147	147	147	147

Analysis uses OLS regression. Dependent variable is contributions. Significantly different from 0 at * 0.1; ** 0.05; *** 0.01. Robust standard errors in parentheses. Variable "# Villages $\leq$ 1.75 km" has been standardized. Controls includes all remaining variables found in Table Controls includes all remaining variables found in Table E1.

Table E8: Effect of Number of Neighbors Within 1.75 km (Control Only)

	(1)	(2)	(3)
# Villages $\leq$ 1.75 km	1.585	−2.327	3.318
	(6.174)	(7.172)	(8.529)
Distance to base (km)		−0.943	−6.581*
		(1.007)	(3.882)
Distance to paved road (km)		3.357	0.785
		(2.339)	(5.161)
Village Size (# HHs)		−0.009	0.162
		(0.119)	(0.160)
Years of Education		1.002	−4.189
		(5.767)	(7.399)
Female		96.928*	63.157
		(53.756)	(69.610)
Age		−8.572	−6.640
		(36.322)	(47.821)
Controls		✓	✓
Sector Fixed Effects			✓
R^2	0.00	0.19	0.47
Observations	74	74	74

Analysis uses OLS regression. Dependent variable is contributions. Significantly different from 0 at * 0.1; ** 0.05; *** 0.01. Robust standard errors in parentheses. Variable "# Villages $\leq$ 1.75 km" has been standardized. Controls includes all remaining variables found in Table Controls includes all remaining variables found in Table E1.

F Counterfactual Planner For Main Treatment

In this section I discuss additional robustness exercises, which will make use of hypothetical logistical paths for the planner to get a sense of how unlikely it would be to observe the results of the paper for the main treatment (defining past neighbor as being within 1.75 km).

First in Section F.1, I consider a number of "worst case scenarios". I imagine scenarios where the logistical planner chooses the ordering in an extreme way – by simply ordering villages according to values of the available variables, and examine the resulting non-significant placebo treatment outcomes.

Second, in Section F.2 I simulate 10,000 counterfactual paths, defining the analogous treatment, and then examining how often an estimate is observed that exceeds the estimates of this paper. Because villages were visited largely sector by sector, I require that the sector order be preserved, but that within sectors, villages are randomly re-ordered. This provides a statistical sense of how exceptional it would be to find the main treatment effects (over 99% of these counterfactual paths result in estimates below those of the paper).

As a final robustness exercise, in Section F.3 I take a slightly different approach. There I use a subset of hypothetical paths to generate a set of instrumental variables for the treatment. I then instrument the observed treatment variable using these deterministically generated instruments, in a two-stage least squares (2SLS) analysis. I find that the 2SLS estimates are positive and significant, and although slightly larger, they are consistent with the matching and OLS estimates.

F.1 Hypothetical Paths I

Here I consider hypothetical village visit orderings which are extreme in the following sense – villages are ordered according to values of the available variables. For example, one scenario involves the planner choosing villages in order from the nearest to the study base to farthest. I do this for all variables (except one) available to the planner, ranking variables from either low to high and vice-versa (randomizing the order with ties).[13] The results of this exercise are shown in Table F1. As one can see, even in these extreme scenarios, the treatment effects are never significant and vary widely and inconsistently across the matching and OLS specifications.

[13]The density of villages within 1.75 km is omitted from this analysis because of insufficient variation. In particular, ties between villages need to be broken randomly which results in variation in these estimates tied to this randomness.

Table F1: Counterfactual Tests

	(1) Matching	(2) Exact Matching	(3) OLS
Distance to base (km)	−14.595	10.899	−19.994
	(19.690)	(19.412)	(15.313)
Reverse order	−0.614	−8.614	−13.318
	(16.136)	(20.579)	(15.438)
Distance to paved road (km)	−6.053	−12.218	−7.393
	(12.278)	(17.252)	(12.774)
Reverse order	−19.049	18.010	−11.854
	(15.451)	(16.379)	(14.043)
Village Size (# HHs)	−7.927	−6.754	−16.916
	(19.372)	(16.794)	(14.227)
Reverse order	−15.956	−9.320	−5.416
	(15.999)	(15.208)	(14.303)

Average Effect of Presence of Past Participating Neighbors, for counterfactual orders of village visits. Dependent variable is contributions. Order of visit is simulated for low to high values for odd rows, and the reverse (high to low values) for even rows. Analysis uses matching and OLS regression, following the empirical strategies in the main paper and this Online Appendix, respectively. Significantly different from 0 at * 0.1; ** 0.05; *** 0.01. Abadie-Imbens or standard robust standard errors in parentheses respectively. Observations vary.

F.2 Hypothetical Paths II

In this section I simulate 10,000 counterfactual paths, calculate the resulting hypothetical treatment (using the 1.75 km distance cut-off), and re-estimate the main analysis of this paper for each path. The construction of these paths adheres closely to the observed pattern of the planner largely proceeding sector by sector. To construct these paths I start with the true village ordering, noting how many villages participated for each sector on each day. Then, holding the sector constant, for each day I randomly replace each village with another in the same sector (without replacement). Note that this type of counterfactual simulation is not the same as the permutation test where treatment is randomly re-assigned and exact tests of significance can be conducted. The reason is that treatment is not a monotonic function of visit order, and also depends on the spatial relationship between villages.

Figures F1 presents the analogous figures for the (a) standard and (b) exact matching strategies, respectively, analogous to the results presented in Figures 2a and 2b in the main paper respectively. From these figures, one can see that the estimates in the main paper exceed 99.92% of the estimated ATEs for the hypothetical treatments for standard matching, and 99.21% of the estimated ATEs for exact matching on sector.

Additionally, Figure F2 presents the results for the distribution of coefficients on these counterfactual treatments, using the OLS strategy in column 3 of Table E1. The estimates in the main paper exceed 98.14% of the estimated coefficients in these counterfactual treatments.

As a further robustness check, I also conducted simulations assuming 10,000 purely random paths. These results, available on request, show that the estimates of this paper exceed 99.9% of these simulated estimates.[14] Overall these simulations suggest that the results of the

[14] I performed one further counterfactual simulation exercise: taking residuals from the regression in Table B1, multiplying them by (-1) with 50% chance, re-assembling the fitted values from the regression with these new residuals, and calculating a new order based on these values. The results show that the estimates of this paper exceed 99.59% of those for standard matching, 97.66% for exact matching, and 96.09% for OLS. I thank an

main paper would be exceptionally rare to occur by chance.

Figure F1: Distribution of Matching Estimates in Counterfactual Treatments

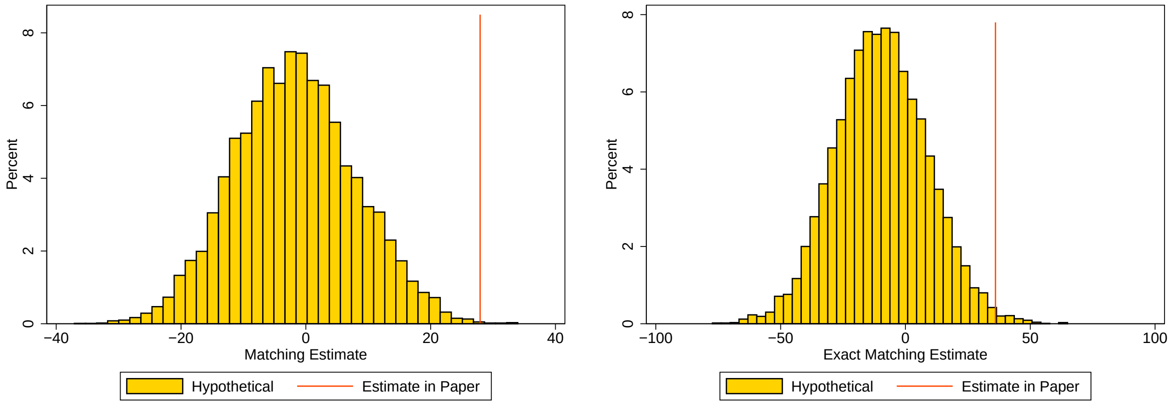

(a) (b)

Distribution of 10,000 estimated ATE estimates from the (a) standard matching and (b) exact matching specifications of the paper, using counterfactual treatments generated from 10,000 random orderings of villages.

Figure F2: Distribution of OLS Coefficients in Counterfactual Treatments

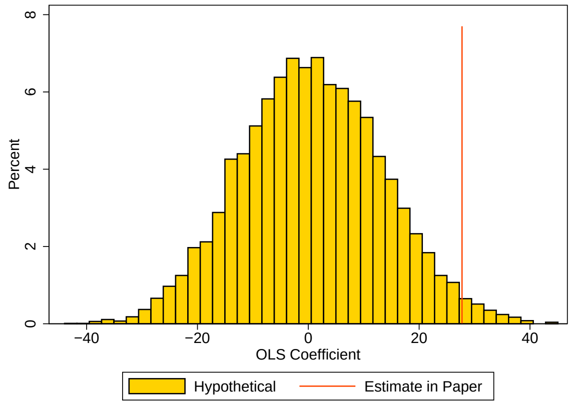

Distribution of 10,000 estimated coefficients from the main OLS specification of this Online Appendix, using counterfactual treatments generated from 10,000 random orderings of villages.

F.3 Instrumental Variable Analysis

The aim of the exercise presented here is to find a set of instruments which are correlated with the main treatment variable, but are otherwise exogenous in relation to contributions in the public goods game. To find such instruments, I simulate six hypothetical routes, using the three observables available to the planner, in increasing and decreasing order (as in Table F1). The hypothetical ordering of villages is conducted as follows. I first rank the 18 political sectors along the dimensions of these three variables (low to high, and vice-versa), taking the average value of the variable per sector. I mandate that the order of visit follow this sector

anonymous referee for this suggestion.

ranking, such that a sector is visited in its entirety, before proceeding to the next sector in the ranking. Within each sector, the order of village visits follows the ranking of the variable under consideration.[15] The next step is to define the number of hypothetically treated neighbors (within 1.75 km), which is the instrument. Because the actual route was chosen principally based on logistical convenience (making use of these observables), and proceeded largely sector by sector, it is reasonable to hypothesize that this instrument will be correlated with the treatment derived from the actual route.

This process generates six potential instruments. To select a set of "optimal" instruments, I follow the post-double selection method of Belloni et al. (2012), which makes use of methods for sparsity, Lasso and Post-Lasso, to select the optimal instruments. The results of these methods select three instruments: (1) the number of participating neighbors generated by the hypothetical ordering which orders sectors (and then villages within sectors) according to their distance to a paved road (from low to high); (2) and (3) the analogous instruments which derive from an ordering by distance to the team's base location, from low to high and from high to low.[16]

Table F2 presents the 2SLS regressions using identical covariates as the primary OLS analysis, Table 3, in the main paper. The estimated coefficients are statistically significant at the 5% level, and significantly larger in magnitude than the matching or OLS estimates. They suggest an effect of communication of about 51 RWF, or a 20% increase in contributions. One note of caution is that these estimates are less precisely estimated, as the standard errors are substantially larger. I cannot reject that the 2SLS estimate is equal to the corresponding OLS coefficient in the main paper, for column 3.[17]

[15]Without imposing such sequentiality, the resulting number of hypothetically treated neighbors is uncorrelated with the treatment, invalidating its use as an instrument. To give an example, one variable is distance to the nearest paved road (ranked low to high). The first sector visited would be the sector with the lowest average distance to the nearest paved road. The first village to be visited would be the village within that sector nearest to a paved road. The following villages within that sector would be visited according to how far they are from a paved road, from shortest to longest. After all villages in the first sector are visited, the second sector chosen is the one with the second lowest average distance to the nearest paved road. This process continues until all villages are visited.

[16]In order to not violate the exclusion restriction, these two variables: distance to a paved road and distance to base must be included in the 2SLS regression.

[17]A final note is that one explanatory variable, the density within 1.75 km is replaced by the density within 3 km, due to collinearity with the derived instrumental variables. Without this modification the results (available on request) are consistently large, with coefficients on treatment status across columns 1 to 3 equaling approximately 49, 65, and 91. However, the instruments are too weak to make any serious inference, and the latter two coefficients are not significant at conventional levels.

Table F2: IV-2SLS - Effect of Presence of Past Participating Neighbors Within 1.75 km

	(1)	(2)	(3)
Treatment Status	49.478*	52.279**	50.611**
	(25.582)	(24.822)	(21.585)
Distance to base (km)	1.951	−1.039	−1.966
	(1.660)	(0.943)	(2.988)
Distance to paved road (km)	0.113	4.538***	1.910
	(0.786)	(1.554)	(4.108)
Village Size (# HHs)		0.041	0.121
		(0.108)	(0.125)
# Villages $\leq$ 3 km		−4.539	−2.668
		(3.004)	(3.195)
Years of Education		1.812	−0.224
		(4.806)	(5.761)
Female		99.634***	96.064**
		(35.443)	(39.378)
Controls		✓	✓
Sector Fixed Effects			✓
First stage F-Statistic	21.12	12.83	11.94
R^2	0.05	0.19	0.28
Observations	147	147	147

Analysis uses instrumental variable two-stage least squares (IV-2SLS) regression. Dependent variable is contributions. See text for instrument selection. Significantly different from 0 at * 0.1; ** 0.05; *** 0.01. Robust standard errors in parentheses. Controls includes all remaining variables found in Table E1.

G Details of Spillovers Across 'Treatment' Versions

This section presents more detailed analysis to Section 3.2 in the main paper. The primary evidence shows that communication spillovers can bias the measurement of preferences in a single version of a public goods game which was played by all villages in the first round. However, many lab-in-the-field studies aim to investigate the impact of different game versions. A possibility is that communication spillovers could impact different versions equally, and thus not lead to bias in estimation of the resulting 'treatment' effects. As outlined in the main paper, to investigate this I make use of the second round of public goods games which utilized one of four different versions, which were selected randomly.

The first version, played in 52% of villages, was the standard baseline game repeated. Note that this version was intentionally over-sampled. The second version, played in 17% of villages, involved punishment. Specifically, after making contributions, each player was anonymously matched with another player whose contribution they saw, and could decide whether to give a penalty. The penalty would remove 100 RWF from the earnings of the matched player, and would cost 50 RWF to the punishing player. The third version, played in 15% of villages, was an analogous reward version, where players could add 100 RWF to a matched player, at a cost of 50 RWF. The fourth version, played in 16% of villages, involved aggregate risk. Rather than having the group fund multiplied by a factor of 3, individuals were given two coin purses and told there were two funds: after all contributions had been made in either (or both) purses, a coin would be flipped to determine which fund would be multiplied by a factor of 5, with the other fund remaining constant. This introduced aggregate risk, although the expected value of contributing was identical. More details can be found in participant instructions in Section A.3.

Given that there are few villages playing the three alternate versions, ranging from 22 to 25, I first pool these versions together, defining the dummy variable *Alt. Version*. I then estimate whether the impact of the alternate versions, compared to the baseline version, was different depending on whether a village had past participating neighbors within 1.75 km (i.e. main treatment status). To do so I examine the same OLS specification found in Table E1, with the addition of the alternate version variable and its interaction with treatment status.

Table G1 presents this specification. Consistent with corresponding Figure 3 in the main paper, the impact of the alternate version differs depending on treatment status. Specifically, while the alternate version has a large and significant positive effect in villages which were unlikely to have had communication spillovers (control), the interaction with treatment status is also large and significant, but negative. Thus, while control villages significantly increased their contributions in alternate versions of the game relative to the baseline, there was no impact in treatment villages. As treatment status is positive and significant, this shows that treatment villages which had past participating neighbors (likely affected by communication spillovers) had overall elevated levels of contributions, independent of whether the version was baseline or not. By contrast, control villages had lower levels of contributions for the baseline version, which substantially increased in alternate versions.

Overall these results show that communication spillovers can impact estimated effects across different versions. Although not shown here, if one was not aware of spillovers and did not account for them, the estimated overall impact of the alternate versions in the column 3 specification would be 19 RWF, and not significant (p-value 0.215). Thus, although the true hypothetical impact among a set of villages which were not subject to communication spillovers appears to be large and positive, ignorance of these spillovers would lead one to erroneously conclude that there is no effect.

For completeness, Table G2 presents the analogous specification separating alternate versions into the three possible versions: penalty, reward, and aggregate risk. One can see that the patterns are similar, though not always significant.

Table G1: Effect of Presence of Past Participating Neighbors Within 1.75 km (Second Round)

	(1)	(2)	(3)
Treatment Status	54.378***	47.057***	38.907**
	(16.659)	(16.644)	(18.860)
Alt. Version	50.104***	48.951***	44.484**
	(13.956)	(17.380)	(19.072)
Alt. Version $\times$ Treatment	-61.274***	-56.867***	-50.565**
	(20.890)	(21.548)	(23.331)
Distance to base (km)		0.419	2.383
		(0.867)	(3.153)
Distance to paved road (km)		3.485*	0.703
		(1.840)	(4.074)
Village Size (# HHs)		-0.054	-0.071
		(0.115)	(0.137)
# Villages $\leq$ 1.75 km		3.208	4.722
		(4.735)	(5.203)
Years of Education		3.018	3.178
		(5.137)	(6.425)
Female		65.810*	47.863
		(38.412)	(44.301)
Controls		✓	✓
Sector Fixed Effects			✓
R^2	0.11	0.23	0.30
Observations	147	147	147

Analysis uses OLS regression. Dependent variable is second round contributions. Significantly different from 0 at * 0.1; ** 0.05; *** 0.01. Robust standard errors in parentheses. Controls includes all remaining variables found in Table E1. Alt. Version is indicator for whether second round was one of three alternate versions: reward, penalty, or aggregate risk.

Table G2: Effect of Presence of Past Participating Neighbors Within 1.75 km (Second Round)

	(1)	(2)	(3)
Treatment Status	54.378***	47.974***	39.713**
	(16.897)	(16.814)	(19.283)
Penalty Version	59.838***	55.445***	50.182**
	(18.549)	(21.379)	(23.086)
Penalty × Treatment	−68.524***	−65.904**	−58.404*
	(26.324)	(28.326)	(29.903)
Reward Version	65.073***	71.353***	65.160***
	(16.660)	(22.780)	(23.432)
Reward × Treatment	−56.297*	−54.855*	−43.373
	(30.837)	(33.338)	(35.863)
Risk Version	22.270	21.851	18.641
	(19.727)	(20.133)	(25.156)
Risk × Treatment	−51.076**	−43.332*	−43.097
	(25.250)	(24.583)	(28.754)
Distance to base (km)		0.358	2.089
		(0.891)	(3.189)
Distance to paved road (km)		3.895**	1.588
		(1.873)	(4.208)
Village Size (# HHs)		−0.033	−0.042
		(0.113)	(0.137)
# Villages ≤ 1.75 km		2.379	3.506
		(4.892)	(5.427)
Years of Education		3.603	4.112
		(5.280)	(6.530)
Female		55.528	34.610
		(37.427)	(44.503)
Controls		✓	✓
Sector Fixed Effects			✓
R^2	0.14	0.26	0.33
Observations	147	147	147

Analysis uses OLS regression. Dependent variable is second round contributions. Significantly different from 0 at * 0.1; ** 0.05; *** 0.01. Robust standard errors in parentheses. Controls includes all remaining variables found in Table E1. Reward, penalty, and risk refer to the different second round game versions – see text.

H Mechanisms: Conditional Cooperation

As noted in the main paper, an individual is classified as a conditional cooperator (CC) if they contribute in the second round the nearest allowable amount to the modal contribution in the first round. If they contribute any other amount, I code them as not being a CC. Because the purses were emptied one by one and counted in front of all participants, the complete distribution of contributions was public knowledge. This procedure makes the mode particularly salient, though it will also be important to compare results when alternative definitions of CC using the median or mean are used, as the next section does.

Aggregating this variable to the village level, the average proportion of CCs is 0.34 (median 0.27), ranging all the way from 0 to 1 in the sample of villages. As noted, it is important to examine whether this proportion does not vary across the treatment and control villages. This difference is not statistically significant at conventional levels, although a relatively low p-value (Ranksum p-value 0.1424) reinforces the exploratory nature of this analysis.[18]

Bearing in mind the caveats of this exercise, to examine the effects of the proportion of CCs on first round contributions, I examine interaction effects with the treatment using an OLS specification.[19] As one can see from Table H1, the effect of the treatment disappears, while the interaction is large and positively significant. The average effect given that villages have on average 34% CCs is approximately 25 RWF. Additionally, CCs contribute significantly greater amounts in general, approximately 99 RWF, or 39% more than their non-CC counterparts. This is intuitive, as typically after CCs, the largest proportion of classifiable subjects are free-riders (Chaudhuri, 2011).

These results correspond to Figure 4 in the main paper, noting that the corresponding OLS regression [not shown here] uses a dummy variable for CCs depending on whether the proportion of CCs is larger than the median (0.27) or not.

[18]I thank two anonymous referees for flagging this potential concern. Given this p-value, I cannot completely rule out the possibility that treatment itself led to more conditionally cooperative behavior. Typical discussion of CCs refers to them as fixed behavioral types. Yet, one could imagine that different types of communication could impact one's willingness to alter their strategy towards a more conditionally cooperative one (Chaudhuri et al., 2006). Regarding what conditional cooperators actually condition on, Hartig et al. (2015) find significant heterogeneity, but that subjects react positively to information on individual contributions, particularly when these contributions are greater and when there is less variation. This also hints at an important role for the mode of the distribution. Chaudhuri (2011) notes that experimental studies have found between 35% and 81% of subjects are CCs, but differences in classification procedures make it difficult to compare.

[19]A previous version of this paper (Coutts, 2019) conducted an analogous matching analysis, however with sample sizes ranging from 20-60, there is not sufficient power to draw sharp conclusions, though the pattern of results is consistent.

Table H1: The Role of Conditional Cooperators (CCs)

	(1)	(2)	(3)
Treatment Status	−2.483	−2.303	−8.588
	(11.823)	(11.420)	(14.273)
Conditional Cooperators (CCs)	114.607***	100.614***	99.142***
	(28.337)	(27.156)	(25.335)
CCs × Treatment	62.649**	67.700**	74.004**
	(31.723)	(30.203)	(31.592)
Distance to base (km)		−0.315	−3.512
		(0.644)	(2.515)
Distance to paved road (km)		1.788	0.737
		(1.266)	(3.735)
Village Size (# HHs)		−0.036	0.052
		(0.098)	(0.107)
# Villages $\leq$ 1.75 km		−2.317	0.804
		(3.184)	(3.929)
Years of Education		0.474	−1.035
		(4.143)	(4.690)
Female		51.919*	26.723
		(28.845)	(32.862)
Controls		✓	✓
Sector Fixed Effects			✓
R^2	0.45	0.51	0.59
Observations	147	147	147

Analysis uses OLS regression. Dependent variable is contributions. Significantly different from 0 at * 0.1; ** 0.05; *** 0.01. Robust standard errors in parentheses. Controls includes all remaining variables found in Table E1. CCs refers to the proportion of individuals classified as conditional cooperators within each village.

H.1 Alternative definitions of CCs

Table H2 presents the effect of CCs for different classifications. Column 1 replicates the result in Table H1 (column 3) above, defining an individual as a CC if they contribute in round 2 the mode of contributions in round 1. Column 2 uses the median, while column 3 uses the mean. From the table, one can see that mode and median results are consistent, while the mean results are not significant. However, the coefficient on the interaction for the mean definition is of the same magnitude, though the estimates are noisier.

Table H2: Robustness: Different definitions of CCs

	(1) Mode	(2) Median	(3) Mean
Treatment Status	−8.588	−23.841	−6.061
	(14.273)	(16.583)	(18.825)
Conditional Cooperator (CC)	99.142***	84.454***	−16.087
	(25.335)	(30.363)	(56.462)
CC × Treatment	74.004**	103.418**	109.275
	(31.592)	(40.897)	(67.412)
Distance to base (km)	−3.512	−3.366	−2.213
	(2.515)	(2.723)	(3.126)
Distance to paved road (km)	0.737	−0.165	−0.538
	(3.735)	(3.728)	(4.393)
Village Size (# HHs)	0.052	−0.056	0.055
	(0.107)	(0.118)	(0.126)
# Villages ≤ 1.75 km	0.804	0.806	1.423
	(3.929)	(4.483)	(5.457)
Years of Education	−1.035	0.264	−0.845
	(4.690)	(5.033)	(6.313)
Female	26.723	32.316	86.786**
	(32.862)	(37.217)	(43.473)
Age	3.190	8.311	11.786
	(18.490)	(18.503)	(24.598)
Controls	✓	✓	✓
Sector Fixed Effects	✓	✓	✓
R^2	0.59	0.53	0.35
Observations	147	147	147

Analysis uses OLS regression. Dependent variable is contributions. Significantly different from 0 at * 0.1; ** 0.05; *** 0.01. Robust standard errors in parentheses. CC refers to the proportion of individuals classified as conditional cooperators within each village. Specifications analogous to Table H1 column 3.

H.2 Alternative Mechanisms

This section critically examines two further alternative mechanisms which could explain the results of increasing contributions.

H.2.1 Time, Field Team Experience, and Contributions

One possible concern is that the field team conducting the experiment gained experience over time, and this altered contributions among later participants relative to earlier participants. This could be a threat to identification of the treatment effects if there were a positive correlation between experience and contributions. Note however that, a priori, there does not seem to be any reason why the effect of experience would be positive or negative. In order to assess the potential for this concern to bias results, I examine the relationship of contributions over time (date of visit), by treatment and control groups separately. Date of visit ranges from 1 to 78.

Figure H1 presents Epanechnikov kernal-weighted local polynomial smoothing plots regarding the relationship between date of visit and contributions. In fact in the main treatment group (villages with previous participating neighbors within 1.75 km) there is only a marginal increase in contributions over time, not statistically significant. This assuages concerns that the treatment effect is driven by increases in contributions due to field team experience, rather than due to communication with past participants. With the corresponding control group (no past

participating neighbors) there do appear some patterns of increasing contributions over time, though this is non monotonic. This alludes to the possibility that some villages in the control group may nonetheless have had contact with previous participants.

Figure H1: Contributions over time

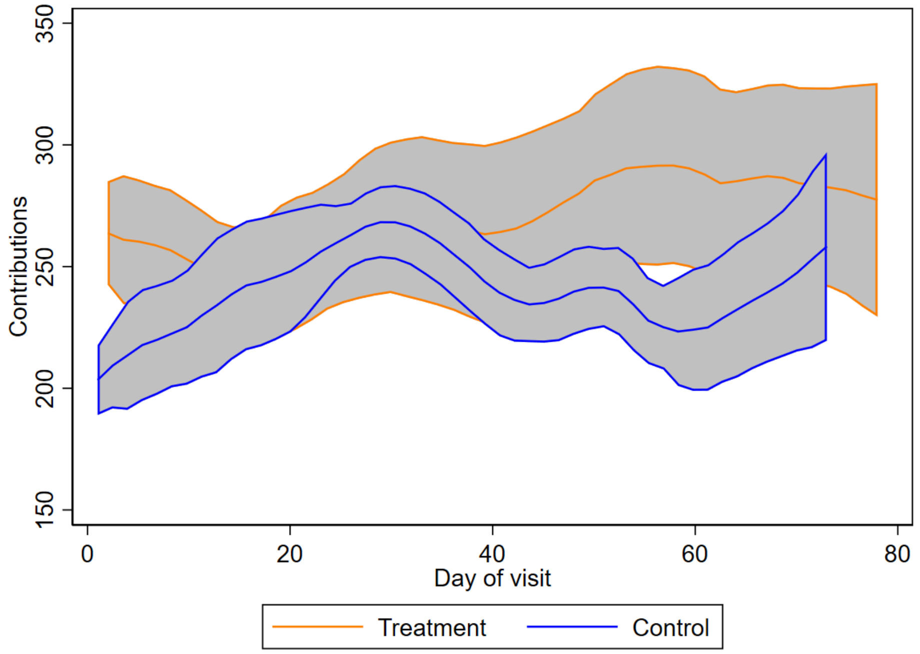

Epanechnikov kernal-weighted local polynomial smoothing plot showing relationship between date of visit and contributions.

Although visit date and treatment status are not significantly associated, they are positively correlated (F-test p-value 0.125), as one would expect given that villages visited later are more likely to have previous participating neighbors. One concern is that some of the observed treatment effect on contributions is being driven by a time trend, itself weakly correlated with treatment. To investigate this concern, first Table H3 presents the matching analysis with the inclusion of date of visit as a match variable. From this table one can see that the estimated average treatment effects are similar to the estimates in the main paper.

One can also add date of visit as a further explanatory variable in the OLS regression. Table H4 presents the analogous regression to Table E1, but with the addition of the date of visit. Date of visit is significant at the 10% level in column 1, but is no longer significant when further controls are added. and does not substantively alter the coefficient on the treatment dummy comparing with the estimates of Table E1.

Table H3: ATE of Presence of Past Participating Neighbors Within 1.75 km

	(1) Standard Matching	(2) Exact Matching
ATE	25.418*	32.761***
	(13.997)	(10.836)
Observations	117	103

Analysis uses nearest neighbor propensity score matching, with 2 neighbors, with replacement. Significantly different from zero at * 0.1; ** 0.05; *** 0.01. Abadie-Imbens Robust Standard Errors in parentheses. Values of propensity score outside common support range are dropped. Exact matching excludes sectors with only 0 or 1 village in either treatment or control groups. Same matching strategy as in main paper, but adds the additional matching variable of day of visit.

Table H4: Effect of Presence of Past Participating Neighbors Within 1.75 km

	(1)	(2)	(3)
Treatment Status	27.961***	25.120**	23.977**
	(10.323)	(10.289)	(11.742)
Date of Visit	0.399*	0.511	0.862
	(0.230)	(0.333)	(0.737)
Distance to base (km)		−0.396	−1.465
		(0.842)	(3.201)
Distance to paved road (km)		2.951	0.753
		(1.907)	(4.459)
Village Size (# HHs)		−0.011	0.077
		(0.117)	(0.138)
# Villages $\leq$ 1.75 km		1.016	3.634
		(4.753)	(5.626)
Years of Education		2.276	1.073
		(4.987)	(6.613)
Female		93.457***	82.731*
		(36.240)	(42.794)
Age		16.436	18.084
		(25.642)	(25.809)
Age^2		−0.218	−0.250
		(0.355)	(0.359)
Controls		✓	✓
Sector Fixed Effects			✓
R^2	0.07	0.22	0.30
Observations	147	147	147

Analysis uses OLS regression. Dependent variable is contributions. Significantly different from 0 at * 0.1; ** 0.05; *** 0.01. Robust standard errors in parentheses. Controls includes all remaining variables found in Table E1.

H.2.2 Potential Changes in Cooperation Norms

Related work by Cardenas and Carpenter (2005) and Bernal et al. (2016) has highlighted the potential pedagogical effect of experimental games, which could positively affect cooperative norms. To investigate whether communication altered norms in this context, Table

H5 examines whether the main treatment, having past participating neighbors within 1.75 km, had any impact on the response to the question, “People in this community generally cooperate with one another on issues that affect the community”. This question was asked before the public goods game was introduced. From this table it is evident that responses to this question were not affected, providing some evidence that community norms about cooperation were not significantly altered. Given the main results in the paper, it appears more plausible that communication changed beliefs specific to behavior in the game, rather than norms in general.

Table H5: Effect of Presence of Past Participating Neighbors Within 1.75 km on Cooperation Norms

	(1)	(2)	(3)
Treatment Status	−0.013	−0.016	−0.008
	(0.022)	(0.021)	(0.019)
Distance to base (km)		−0.001	0.002
		(0.001)	(0.006)
Distance to paved road (km)		0.000	−0.007
		(0.002)	(0.007)
Village Size (# HHs)		0.000	0.000
		(0.000)	(0.000)
# Villages $\leq$ 1.75 km		0.013	0.014*
		(0.008)	(0.008)
Years of Education		−0.006	0.003
		(0.008)	(0.009)
Female		0.013	0.110*
		(0.062)	(0.066)
Age		0.057*	0.062*
		(0.033)	(0.034)
Age^2		−0.001*	−0.001**
		(0.000)	(0.000)
Controls		✓	✓
Sector Fixed Effects			✓
R^2	0.00	0.44	0.58
Observations	147	147	147

Analysis uses OLS regression. Dependent variable is *Community Cooperation Index*. Significantly different from 0 at * 0.1; ** 0.05; *** 0.01. Robust standard errors in parentheses. Controls includes all remaining variables found in Table E1.

I Time and the Window of Communication

Of particular interest is the relationship between time elapsed since neighboring villages' participation and the contributions of treatment villages. Importantly, this relationship is ambiguous: more time between visits could allow information to reach more individuals, or it could lead the information to decay due to lost interest or lower salience. The median of the average number of days elapsed since a treatment village's neighbors participated is 5 days, with a minimum of 1 and a maximum of 61. To explore this, I divide the treatment villages into three terciles: short ($<$ 3 days), medium (3-7 days), and long ($>$ 7 days). Table I1 presents the analog of the main OLS regression in Table E1, separating the treatment villages into these

three groups. From Table I1, the strongest effects appear to be driven by the intermediate timing, of 3 to 7 days, showing a large effect that increases contributions by 18%. Effects are also visible for shorter durations, but these are not always statistically significant. Finally, for villages which had neighbors that participated one week or more earlier, there is little evidence for strong effects, though the coefficient remains positive. While these results are suggestive that communication spillovers will have the largest impacts for intermediate dates (here 3-7 days). The lowest effects are seen for longer durations of more than 7 days, suggesting that having a long enough cooling-off period could reduce the impact of spillovers. However, one should take into account the data limitations from such ex-post divisions of the treatment group.[20]

Table I1: Effect of Presence of Past Participating Neighbors Within 1.75 km (By time elapsed)

	(1)	(2)	(3)
Treatment Status (short)	22.493*	22.613*	21.780
	(13.088)	(13.488)	(14.705)
Treatment Status (medium)	49.005***	45.648***	46.757**
	(17.892)	(16.384)	(18.870)
Treatment Status (long)	17.656	14.092	14.065
	(15.550)	(15.823)	(20.177)
Distance to base (km)		−0.202	−0.695
		(0.859)	(3.102)
Distance to paved road (km)		3.960**	0.793
		(1.652)	(4.202)
Village Size (# HHs)		−0.000	0.093
		(0.114)	(0.141)
# Villages $\leq$ 1.75 km		−0.070	3.069
		(4.642)	(5.364)
Years of Education		2.621	−0.162
		(4.787)	(6.364)
Female		100.870***	86.224**
		(37.743)	(42.528)
Controls		✓	✓
Sector Fixed Effects			✓
R^2	0.08	0.22	0.31
Observations	147	147	147

Analysis uses OLS regression. Dependent variable is contributions. Significantly different from 0 at * 0.1; ** 0.05; *** 0.01. Robust standard errors in parentheses. Controls includes all remaining variables found in Table A1. Short: average date of neighbors' participation was < 3 days, medium: $3 - 7$ days, and long: > 7 days.

J Discussion of previous lab-in-the-field studies

The introduction of the main paper referenced 10 studies in leading economics and political science journals published between 2015-2018 which involved lab-in-the-field experiments, as well as 7 studies that were identified in the top field journal *Journal of Development Economics* from 2010-2019 using google scholar keyword searches (for "lab-in-the-field" and

[20]Coutts (2019) examined this table for the alternative distance of 2 km, showing that in this alternative specification both short and intermediate durations lead to significant large effects. Longer durations remain non-significant.

“artefactual”).[21] One note is that the majority of these studies examine aggregate population behavior, or relate lab-in-the-field behavior to real-world outcomes. A minority of studies examine within-game treatments, which may still be susceptible to bias from communication spillovers, as noted in Section 3.2 of the main paper. Several of these studies also include some component which involves a comparison of behavior with real-world outcomes.

Regarding the first ten studies, six do not mention possible spillovers, while for between three and four of the ten one could potentially be concerned about spillovers. Specifically, as part of a field experiment, Casaburi and Macchiavello (2018) conduct lab-in-the-field experiments with farmers from a cooperative with 2,000 members and local traders, but do not mention the effect of timing or location of the experiments.[22] Gneezy et al. (2016) conduct lab-in-the-field games in Brazil, showing the general area and the general time-frame, but do not show specific details about timing or session locations. Kosfeld and Rustagi (2015) conduct their games with 56 different forest user groups, in five villages in Ethiopia. Appendices provide detailed information about strategies to try and mitigate communication spillovers, due to some forest groups being geographically close.[23] Bauer et al. (2018) conduct their experiments in 33 villages in Uganda, and while they paid careful attention spillovers across games within villages, they do not mention spillovers across villages (from their map it appears some of their villages are located within 2 km of another sampled village).[24] Somville and Vandewalle (2018) conduct their lab-in-the-field games in 18 villages in India, and do not mention spillovers, although distance suggests they may not have been a concern.[25] Chandrasekhar et al. (2018) note that their villages are located far apart, which would preclude spillovers. Enos and Gidron (2018) conduct their experiment with randomly approached participants in cities in Israel, while not mentioning spillovers, their approach likely precludes such spillovers. Avdeenko and Gilligan (2015) mention geographical isolation to rule out spillovers. D’Exelle and Verschoor (2015) explicitly mention strategies to reduce spillovers in Uganda: namely conducting all experiments in the same sub-county on the same day.[26] Jakiela and Ozier (2015) ensure communities are 5 km apart, to avoid spillovers.

Regarding the seven studies identified in the *Journal of Development Economics*, they were published between 2012 and 2019. Between four and five raise some concerns that spillovers could have affected the results, and do not provide details, in published materials, of whether any precautions were taken to mitigate these threats. Baland et al. (2019) designed and conducted a lab-in-the-field game in eight neighborhoods of Cotonou-Calavi in Benin, between May and June 2018. They conducted 40 sessions (for an average of 5 sessions per neighborhood) in the same community center for each neighborhood, indicating a possible risk of spillovers. Information about session timing does not appear to be explicitly mentioned. Binzel and Fehr (2013) conducted trust and dictator games with residents of an informal

[21]Studies which involved lab-in-the-field experiments focusing only on individual decision making were excluded, as one would expect that communication spillovers should be less of a concern (although this is an empirical question).

[22]In private correspondence the authors noted the (non-survey) lab-in-the-field experiments were conducted at the same location; in one case farmers participated at sparse intervals, in the other participation was jointly in a given day.

[23]Namely, for neighboring groups the authors conducted all sessions without breaks until all groups had participated, to minimize potential interactions between past and future participants.

[24]In private correspondence the authors noted they were concerned about spillovers, and made efforts to schedule neighboring villages on sequential days.

[25]Distance is not referenced in the paper, however in private correspondence the authors noted that the shortest distance between two villages was 3.5 km.

[26]In private correspondence the authors noted they took additional safeguards to ensure no contact between morning and afternoon participants.

housing area of Cairo, in five experimental sessions. The sessions were located in the same cultural theatre and took place over time frame of one-week, indicating some likelihood of communication spillovers. The density and recruitment procedures may have worked to limit the effect of spillovers; the authors note in online supplementary material that participants knew on average 8% of other participants in their sessions.[27] Boltz et al. (2019) conduct lab-in-the-field experiments in seven communities in a department of Dakar, in May-June 2014. Experiments within the same community occurred in the same location, increasing the risk for spillovers, but were conducted within the same day, which likely lowered this risk. Due to the population density in the region, spillovers across communities would likely not be a concern. While spillovers are not explicitly mentioned, the design includes elements which indicate the authors took them into account.

Cárdenas et al. (2013) conduct lab-in-the-field experiments across six Latin American cities. They conducted approximately 25 sessions in each city. Location and timing details for each specific city are not available, which makes it difficult to evaluate the risk.[28] Ligon and Schechter (2012) conducted lab-in-the-field games in fifteen villages in rural Paraguay, at a central location within each village. No mention is made of distance or timing across villages, although given the randomized selection procedure, one would assume they were sufficiently distant from one-another.[29] Czura (2015) conducted lab-in-the-field games with clients of a microlending institution in Bihar, India. Six sessions occurred in the same location, indicating some potential risk for communication spillovers. Information of timing does not appear to be explicitly mentioned.[30] Hill et al. (2012) examine dictator and trust games in eight communities in rural Peru, where some communities participated in more than one session, indicating some risk for spillovers. Distances appear to be moderate to large across communities. Precise information about timing does not appear to be available.[31]

References

Avdeenko, Alexandra and Michael J Gilligan, "International Interventions to Build Social Capital: Evidence from a Field Experiment in Sudan," *American Political Science Review*, 2015, pp. 1–45.

Baland, Jean Marie, Catherine Guirkinger, and Renate Hartwig, "Now or later? The allocation of the pot and the insurance motive in fixed roscas," *Journal of Development Economics*, 2019, *140* (December 2018), 1–11.

Bauer, Michal, Nathan Fiala, and Ian Levely, "Trusting Former Rebels: An Experimental Approach to Understanding Reintegration after Civil War," *Economic Journal*, 2018, *128* (613), 1786–1819.

[27]In private correspondence the authors noted they took additional steps to reduce the possibility of spillovers, including conducting the workshops within a short time frame, recruiting from disparate areas, and requesting participants not to discuss the workshop with others.

[28]Recruitment procedures did ensure that participants in the same session did not know each other.

[29]In private correspondence the authors confirmed this, sharing their distance data, showing that the average distance to the closest village was approximately 31 km, with the shortest distance being approximately 7 km.

[30]In private correspondence with the author, she provided the timing details that sessions were conducted in three consecutive days, with participants on different days coming from different borrowing centers, to limit the risk of spillovers.

[31]In private correspondence the authors noted that mechanics of recruitment were such that participants were typically contacted on the same day of participation, which may have reduced the potential for communication spillovers.

Belloni, Alexandre, Daniel Chen, Victor Chernozhukov, and Chris Hansen, "Sparse Models and Methods for Optimal Instruments With an Application to Eminent Domain," *Econometrica*, 2012, *80* (6), 2369–2429.

Bernal, Adriana, Juan-Camilo Cárdenas, Laia Domenech, Ruth Meinzen-Dick, and Sarmiento Paula J., "Social Learning through Economic Games in the Field," *mimeo*, 2016, pp. 1–33.

Binzel, Christine and Dietmar Fehr, "Social distance and trust: Experimental evidence from a slum in Cairo," *Journal of Development Economics*, 2013, *103* (1), 99–106.

Boltz, Marie, Karine Marazyan, and Paola Villar, "Income hiding and informal redistribution: A lab-in-the-field experiment in Senegal," *Journal of Development Economics*, 2019, *137* (October 2018), 78–92.

Cárdenas, Juan Camilo, Alberto Chong, and Hugo Ñopo, "Stated social behavior and revealed actions: Evidence from six Latin American countries," *Journal of Development Economics*, 2013, *104*, 16–33.

Cardenas, Juan Camilo and Jeffrey P. Carpenter, "Three themes on field experiments and economic development," in John A. List Glenn W. Harrison, Jeffrey Carpenter, ed., *Field Experiments in Economics*, 2005, pp. 71–123.

Casaburi, Lorenzo and Rocco Macchiavello, "Demand and Supply of Infrequent Payments as a Commitment Device: Evidence from Kenya," *American Economic Review*, 2018.

Chandrasekhar, Arun G., Cynthia Kinnan, and Horacio Larreguy, "Social networks as contract enforcement: Evidence from a lab experiment in the field," *American Economic Journal: Applied Economics*, 2018, *10* (4), 43–78.

Chaudhuri, Ananish, "Sustaining cooperation in laboratory public goods experiments: A selective survey of the literature," *Experimental Economics*, 2011, *14*, 47–83.

_ , Sara Graziano, and Pushkar Maitra, "Social Learning and Norms in a Public Goods Experiment with Inter-Generational Advice," *Review of Economic Studies*, apr 2006, *73* (2), 357–380.

Coutts, Alexander, "Identifying communication spillovers in lab in the field experiments," *Novafrica Working Paper No. 1903*, 2019.

Czura, Kristina, "Pay, peek, punish? Repayment, information acquisition and punishment in a microcredit lab-in-the-field experiment," *Journal of Development Economics*, 2015, *117*, 119–133.

D'Exelle, Ben and Arjan Verschoor, "Investment Behaviour, Risk Sharing and Social Distance," *Economic Journal*, 2015, *125* (584), 777–802.

Enos, Ryan D. and Noam Gidron, "Exclusion and Cooperation in Diverse Societies: Experimental Evidence from Israel," *American Political Science Review*, jul 2018, pp. 1–16.

Gneezy, Uri, Andreas Leibbrandt, and John A. List, "Ode to the Sea: Workplace Organizations and Norms of Cooperation," *Economic Journal*, 2016, *126* (595), 1856–1883.

Hartig, Björn, Bernd Irlenbusch, and Felix Kölle, "Conditioning on what? Heterogeneous contributions and conditional cooperation," *Journal of Behavioral and Experimental Economics*, apr 2015, *55*, 48–64.

Hill, Ruth Vargas, Eduardo Maruyama, and Angelino Viceisza, "Breaking the norm: An empirical investigation into the unraveling of good behavior," *Journal of Development Economics*, 2012, *99* (1), 150–162.

Imbens, Guido W., "Matching Methods in Practice: Three Examples," *Journal of Human Resources*, 2015, *50* (2), 373–419.

_ **and Jeffrey M. Wooldridge**, "Recent developments in the econometrics of program evaluation," *Journal of Economic Literature*, 2009, *47* (1), 5–86.

Jakiela, Pamela and Owen Ozier, "Does Africa Need a Rotten Kin Theorem? Experimental Evidence from Village Economies," *Review of Economic Studies*, 2015.

Kosfeld, Michael and Devesh Rustagi, "Leader Punishment and Cooperation in Groups: Experimental Field Evidence from Commons Management in Ethiopia †," *American Economic Review*, 2015, *105* (2), 747–783.

Kremer, M. and E. Miguel, "The Illusion of Sustainability," *The Quarterly Journal of Economics*, aug 2007, *122* (3), 1007–1065.

Ligon, Ethan and Laura Schechter, "Motives for sharing in social networks," *Journal of Development Economics*, 2012, *99* (1), 13–26.

Oster, Emily and Rebecca Thornton, "Determinants of technology adoption: Peer effects in menstrual cup take-up," *Journal of the European Economic Association*, 2012, *10* (6), 1263–1293.

Rosenbaum, Paul R and Donald B Rubin, "The Central Role of the Propensity Score in Observational Studies for Causal Effects," *Biometrika*, apr 1983, *70* (1), 41.

Sinharoy, Sheela S., Wolf-Peter Schmidt, Kris Cox, Zachary Clemence, Leodomir Mfura, Ronald Wendt, Sophie Boisson, Erin Crossett, Karen A. Grépin, William Jack, Jeanine Condo, James Habyarimana, and Thomas Clasen, "Child diarrhoea and nutritional status in rural Rwanda: a cross-sectional study to explore contributing environmental and demographic factors," *Tropical Medicine & International Health*, aug 2016, *21* (8), 956–964.

Sinharoy, Sheela S, Wolf-Peter Schmidt, Ronald Wendt, Leodomir Mfura, Erin Crossett, Karen A. Grépin, William Jack, Bernard Ngabo Rwabufigiri, James Habyarimana, and Thomas Clasen, "Effect of community health clubs on child diarrhoea in western Rwanda: cluster-randomised controlled trial," *The Lancet Global Health*, jul 2017, *5* (7), e699–e709.

Somville, Vincent and Lore Vandewalle, "Saving by default: Evidence from a field experiment in rural India," *American Economic Journal: Applied Economics*, 2018, *10* (3), 39–66.
